# Factor H-related 1 and heparan sulfate architecture contribute to complement dysregulation in C3 glomerulopathy

**DOI:** 10.3389/fimmu.2025.1589674

**Published:** 2025-05-16

**Authors:** Amanda K. Slagle, Nicolo Ghiringhelli Borsa, Kai Wang, Amanda O. Taylor, Nicole C. Meyer, Michael B. Jones, William D. Walls, Angela F. M. Nelson, Sarah M. Roberts, Mingyao Sun, Elena Goicoechea de Jorge, Santiago Rodriguez de Cordoba, Diana I. Jalal, Carla M. Nester, Yuzhou Zhang, Richard J. H. Smith

**Affiliations:** ^1^ Molecular Otolaryngology and Renal Research Laboratories, Carver College of Medicine, University of Iowa, Iowa, IA, United States; ^2^ Graduate PhD Program in Immunology, Carver College of Medicine, University of Iowa, Iowa, IA, United States; ^3^ Department of Biostatistics, College of Public Health, University of Iowa, Iowa, IA, United States; ^4^ Department of Internal Medicine, Carver College of Medicine, University of Iowa, Iowa, IA, United States; ^5^ Centro de Investigaciones Biológicas Margarita Salas, Consejo Superior de Investigaciones Científicas, Madrid, Spain; ^6^ Center for Comprehensive Access and Delivery Research and Evaluation, Iowa City VA Health Care System, Iowa, IA, United States

**Keywords:** Factor H, FHR-1, complement regulation, C3 glomerulopathy, chronic kidney disease

## Abstract

**Introduction:**

Dysregulation of the alternative pathway of complement underlies the pathogenesis of C3 glomerulopathy (C3G). Because Factor H (FH) prevents excessive alternative pathway activity while Factor H-related protein 1 (FHR-1) is believed to enhance this response, we investigated the balance between FH and FHR-1 in C3G.

**Methods:**

To assess the role of FHR-1 in C3G pathogenicity, we used a multiplex ligation-dependent probe amplification to detect copy number variants in *CFHR3-CFHR1* and enzyme linked immunosorbent assays to measure circulating protein levels in C3G patients compared to controls. Additionally, an *in vitro* C3b deposition assay was used to characterize the functional impact of FHR-1 on local complement activity.

**Results:**

In this study, we confirm that *CFHR3-CFHR1* copy number impacts C3G risk. In C3G patients with two copies of *CFHR3-CFHR1*, the FHR-1:FH protein ratios are increased compared to controls; however, this increase is not disease specific. Rather, it is reflective of deteriorating renal function and was also observed in a second cohort of patients with chronic kidney disease from a variety of other causes. Functional studies showed that FHR-1 competes with FH to increase C3b deposition on mouse mesangial cell surfaces, an effect enhanced by heparan sulfate cleavage.

**Discussion:**

Altogether, we show that as renal function declines, a change in the FHR-1:FH ratio combined with changes in heparan sulfate architecture increase complement activity. These findings suggest that complement activity may contribute to the chronic inflammation and progression of renal damage associated with chronic kidney disease.

## 1 Introduction

C3 glomerulopathy (C3G) is a rare form of glomerular disease characterized by C3 deposition on kidney biopsy. Disease pathogenesis is driven by complement dysregulation that leads to end stage kidney disease in 50% of affected patients within 10 years of diagnosis. Two major subtypes are recognized – dense deposit disease and C3 glomerulonephritis – distinguished by differences resolved by electron microscopy. Underlying the complement dysregulation are genetic factors in complement genes of the alternative pathway (AP) and/or autoantibodies to complement proteins or protein complexes, found in 20-25% and 40-60% of patients, respectively (1–7). In 35-45% of patients, however, a cause for complement dysregulation is not identified (8).

One of the major regulators of complement is a protein called Factor H (FH). Encoded by the *CFH* gene, FH is made up of 20 short consensus repeats (SCRs). The first four N-terminal SCRs regulate complement activity while the two C-terminal SCRs are cell recognition and ligand binding domains. Dysfunction or insufficiency of FH is associated with development of several complement-based diseases including C3G (9–14).

Contiguous with *CFH* in the regulators of complement activation gene cluster on chromosome 1q32 are five Factor H-related genes in chromosomal order *CFHR3, CFHR1, CFHR4, CFHR2, CFHR5*. They encode the Factor H-related proteins (FHRs), which exhibit cell surface ligand-binding capabilities similar to FH but lack the N-terminal regulatory domains (15, 16). Notably, the three type 1 FHRs – FHR-1, FHR-2, and FHR-5 – share two N-terminal SCRs that form a dimerization domain, enabling both homodimerization (e.g., FHR-1:FHR-1, FHR-2:FHR-2, FHR-5:FHR-5) and heterodimerization (e.g., FHR-1:FHR-2; FHR-1:FHR-5 and FHR-2:FHR-5 do not heterodimerize) (17–19). Functional studies suggest that these FHRs compete with FH for binding to shared surface ligands thereby promoting AP activation (12, 20, 21).

Currently, it is believed that FHRs and FH maintain a balance between complement activation and regulation on cell surfaces. Disruption of this balance has been associated with specific pathological conditions, including C3G (12, 19–31). Genetic variations linked to C3G, such as gain-of-function gene fusion events involving *CFHR1* and *CFHR5*, which duplicate the dimerization domains of these proteins, highlight the critical role of FHR-1 and FHR-5 in complement control and C3G pathogenesis (21, 32). Further evidence comes from mass spectrometry and immunohistochemistry data of kidney tissue from C3G patients, which consistently identify high levels of FHR-1 and FHR-5 in glomeruli (33, 34).

In this study, we explore the relationship between FH, FHR-1, and FHR-5 in C3G. At the genetic level, we characterize the genotype frequencies of *CFHR3-CFHR1* in C3G patients and at the protein level, we assess circulating levels of FH, FHR-1, and FHR-5 in C3G patients as compared to disease-free controls and patients with other chronic kidney diseases (CKD). Because complement control by FH is mediated in part through its binding to polyanionic molecules such as heparan sulfate (HS) and HS is damaged in chronic kidney disease, we also explore the functional impact of FH and FHR-1/FHR-5 on healthy and heparinase-treated cell surfaces. We hypothesize that C3G patients have elevated levels of FHR-1 and FHR-5, which compromise FH regulation on cell surfaces, and that HS damage impacts the binding profiles of FH, FHR-1 and FHR-5 further affecting complement activity in the glomerular microenvironment and shifting complement regulation in favor of AP activation.

## 2 Materials and methods

### 2.1 Study populations

To evaluate the effect of *CFHR3-CFHR1* copy numbers on development of C3G, 280 patients diagnosed with C3G between the years 2009-2023 (stratified by non-Finnish Europeans (NFE)(n = 241) and African/African Americans (AFR)(n = 39) to remove ethic confounders) were compared to data from the Genome Aggregation Database (gnomAD) structural variants v4.1.0 (gnomad.broadinstitute.org).

To evaluate circulating protein levels, 98 C3G patients with wild-type *CFHR3-CFHR5* and no genetic mutations in disease-associated complement genes were compared to two control groups, 1) 88 persons with normal kidney function and wild-type *CFHR3-CFHR5* and 2) 50 randomly selected patients with stage 3–4 CKD. A description of the CKD patients has been published elsewhere; copy numbers were not assessed (35). Importantly, patients with a history of immune-mediated kidney disease or having received immunosuppressive therapy within the last one year were excluded.

Ethical approval for this study was granted by the Institutional Review Board of the Carver College of Medicine at the University of Iowa.

### 2.2 Copy number variants

*CFHR3-CFHR1* copy number variation was determined by multiplex ligation dependent probe amplification (MLPA) using in-house designed probe pairs and the SALSA MLPA EK20-FAM kit (MRC Holland), as previously described (36–38). In brief, copy number was assigned as two copies (wild type), one copy (single deletion), zero copies (homozygous deletion), or more than two copies (duplications) based on internally validated peak area ratios of 0.7-1.33, 0.25-0.68, 0-0.21, and >1.34, respectively. If ratios were outside of these designated ranges, samples were re-assayed.

### 2.3 FH, FHR-1, and FHR-5 protein levels

Serum concentrations of FH were measured using the Factor H MicroVue Complement kits (Quidel Ortho Corp., San Diego, CA), while FHR-1 and FHR-5 were measured by enzyme-linked immunosorbent assays (ELISA). The FHR-1 ELISA has been described previously (39), while the FHR-5 ELISA was developed in house. In brief, 96-well flat bottom plates (Corning Incorporated, Corning, NY) were coated overnight with a monoclonal FHR-5 antibody (MAB3845 lot #ZFL0419071, R&D Systems, Inc., Minneapolis, MN) diluted in sodium bicarbonate/carbonate coating buffer (pH 9.6) at a final concentration of 5μg/ml. Plates were washed (PBST 0.05%) and blocked for 1h at room temperature (RT) with ELISA Ultra Block Buffer (Bio-Rad, Minneapolis, MN), followed by a 1h incubation with patient serum diluted 1:600 in PBST. Next, samples were incubated at RT for 1h with a polyclonal FHR-5 antibody (PA5–87846 lot #WL3447164B, ThermoFisher Scientific, Inc., Waltham, MA) diluted in PBST followed by a secondary goat anti-rabbit (Kindle Bioscience, San Diego, CA). ELISAs were developed using TMB substrate (SeraCare, Milford, MA) and absorbance was measured at λ450. Standard curves were generated using serial dilutions of recombinant human FHR-5 (rFHR-5)(R&D Systems, Minneapolis, MN) from 1.5 μ g/ml to 0.006 μ g/mL. Results were interpreted by four-parameter logistic regression (www.myassays.com).

### 2.4 C3b deposition assay

C3b deposition was quantified using mouse mesangial cells (MES-13)(ATCC CRL-1927; Manassas, VA) cultured on Lab-Tek II CC^2–^8 well glass chamber slides (ThermoFisher Scientific, Inc., Waltham, MA). MES-13 cells were preincubated at 37°C for 30 minutes with media alone or media supplemented with 10μU/ml heparinase II and 10μU/ml heparinase III (IBEX Pharmaceuticals, Montreal, Canada). Cells were then washed (1XPBS) and incubated at 37°C for 15 minutes with FH-depleted serum (Complement Technologies, Houston, TX) supplemented with 165μg/ml human FH (Complement Technologies, Houston, TX), 10μg/ml recombinant human FHR-1*A (rFHR-1*A)(R&D Systems, Inc., Minneapolis, MN), 10μg/ml recombinant human FHR-1*B (rFHR-1*B)(Sino Biological, Houston, TX), 10μg/ml rFHR-5 (R&D Systems, Inc., Minneapolis, MN) and/or 55.5mg/dl human C3 (Complement Technologies, Tyler, TX) with EGTA and Mg^++^ (0.5mM). Next, cells were washed and fixed for 10 minutes with 2% paraformaldehyde followed by blocking buffer (BSA, glycine, 0.05% PBST) for 30 minutes at RT. After a second 30-minute incubation at RT with primary C3b 7C12 (BioLegend, San Diego, CA) and 10E4 antibodies (Amsbio, Cambridge, MA), cells were incubated with secondary Alexa-488 (Invitrogen, Waltham, MA) and Alexa-568 (Jackson ImmunoResearch, West Grove, PA) antibodies for 30 minutes. Cells were then washed, mounted with ProLong™ Diamond Antifade Mountant with DAPI (Invitrogen, Waltham, MA) and examined using a Leica DMI8 Confocal Microscope. Image analysis was performed using ImageJ (version 1.53s).

### 2.5 Heparan sulfate glycan microarray

Binding capabilities of human FH SCRs 15-20 (R&D Systems, Inc., Minneapolis, MN), rFHR-1*A (R&D Systems, Inc., Minneapolis, MN), and rFHR-5 (R&D Systems, Inc., Minneapolis, MN) to various heparan sulfate (HS) glycans was analyzed using the HS Glycan Microarray from Z Biotech at equal molar concentrations of 0.2µM for direct comparison (**Supplementary Figure 1**). HS glycans (Glycan Therapeutics Inc., Raleigh, NC) were tagged with an aldehyde group at the reducing end and fabricated on multivalent hydrazide slides (Z Biotech, Aurora, CO). For printing the HS Glycan Array, glycans were dissolved in 150 mM sodium phosphate buffer (pH 5.8) at 100μM concentration. Each slide contained 8- or 16-subarrays with each subarray containing 6 replicate spots per glycan. Each glycan was deposited ~1.2nL per spot by no-contact dispensing from Nano-Plotter 2.1 (GESIM) at ambient temperature and relative humidity 50%. Each array was laid out according to the user manual at Z Biotech. For quality control, each batch of the HS Glycan Array products were assayed with plant lectins (GS-II, WGA) and antithrombin III protein. Five controls were selected for this assay: 1) print buffer without glycans, 2) anti-6x Histidine antibody, 3) anti-rabbit IgG (H+L) Cy3 antibody, 4) 6x histidine peptide (APExBIO, Houston, TX) and detection reagents, and 5) rabbit IgG (Invitrogen, Carlsbad, CA). FH, rFHR-1*A and FHR-5 were precomplexed with detection reagents anti-6x Histidine antibody (Invitrogen, Carlsbad, CA) and anti-rabbit IgG (H+L) Cy3 antibody (Life Technologies, Carlsbad, CA) at a ratio of 25:1:1 µg/ml on ice for 1h. The microarray slide (Z Biotech, Aurora, CO) was pretreated with glycan array blocking buffer (GABB)(Z Biotech, Aurora, CO) for 30 minutes at RT. The slide was then washed using GAAB and incubated for 1h at RT with the precomplexed samples. Finally, the slide was washed and scanned at 10μm/pixel with a laser channel of 532nm using an Innoscan 710 microarray scanner (Innopsys, Carbonne, France) and Mapix software (version 9.1.0). The data were processed by data sorting software and a binding motif mining tool (MotifFinder version 3.1.2). Percent of maximum binding was calculated with error bars representing standard deviation from values of each replicate.

### 2.6 Gel electrophoresis and western blot analysis

SDS-PAGE was performed on human serum samples using mini-protein TGX precast 4-15% gels (Bio-Rad, Minneapolis, MA). Various primary antibodies including anti-human C3b [7C12] (BioLegend, San Diego, CA), FH [OX-24] (ThermoFisher Scientific, Inc., Waltham, MA), Factor I [EPR23948-48] (Abcam, Boston, MA), and FHR1/FHR2 (in-house generated) followed by secondary horseradish peroxidase-conjugated goat anti-mouse or anti-rabbit (Jackson ImmunoResearch, West Grove, PA) were used for Western blotting. Membranes were developed with SuperSignal™ West Pico PLUS Chemiluminescent Substrate (ThermoFisher Scientific, Inc., Waltham, MA) following the manufacturer’s instructions. Images were captured using the iBright 1500 (Invitrogen, Waltham, MA).

### 2.7 Statistical analysis

All statistical analyses were performed using RStudio (R version 3.6.1 (2019-07-05))) or GraphPad (version 10) using a variety of tests (Fisher’s exact test, chi-squared statistic, Kruskal-Wallis tests followed by Dunn’s multiple comparison test, receiver operating characteristic curve, or logistic regression model) to assess differences between cohorts. During analysis, a listwise deletion approach was taken to exclude cases with missing data. *P* values < 0.05 were considered statistically significant.

## 3 Results

### 3.1 The *CFHR3-CFHR1* deletion is protective against C3G in NFE

MLPA was used to detect *CFHR3-CFHR1* deletions (del(*CFHR3-CFHR1*)) and duplications (dup(*CFHR1-CFHR3*) in 241 NFE and 39 AFR C3G patients. The distribution of wild type, heterozygous del(*CFHR3-CFHR1*), and homozygous del(*CFHR3-CFHR1*) was 172, 64, and 3 in NFE patients and 15, 19 and 5 in AFR patients. Dup(*CFHR3-CFHR1*) were identified in 2 NFE patients but no AFR patients. As compared to gnomAD controls, the distribution of genotypes in NFE C3G patients was significantly different (*P* = 1.531e-07), with a decrease in del(*CFHR3-CFHR1*) and an increase in dup(*CFHR3-CFHR1*)(**Table 1**). No differences were seen in AFR C3G patients as compared to controls (*P* = 0.5043). These results indicate that deletion of *CFHR3-CFHR1* is protective against C3G in NFEs.

**Table 1:** *CFHR3-CFHR1* genotype frequencies in C3 glomerulopathy patients as compared to controls.

	gnomAD NFE (n = 29543)	C3G NFE (n = 241)	gnomAD AFR (n = 16908)	C3G AFR (n = 39)
Wild Type	0.65 (19184)	0.71 (172)	0.36 (6162)	0.38 (15)
Heterozygous del_(_ **_Δ_** *_CFHR3-CFHR1)_*	0.33 (9822)	0.27 (64)	0.55 (9304)	0.49 (19)
Homozygous del_(_ **_Δ_** *_CFHR3-CFHR1_*_)_	0.018 (537)	0.012 (3)	0.085 (1442)	0.13 (5)
Duplications	0.0 (0)	0.008 (2)	0.0 (0)	0.0 (0)
*P* value	1.531e-07		0.5043	

### 3.2 FHR-1:FH ratios are significantly increased in C3G patients

**Figure 1:**
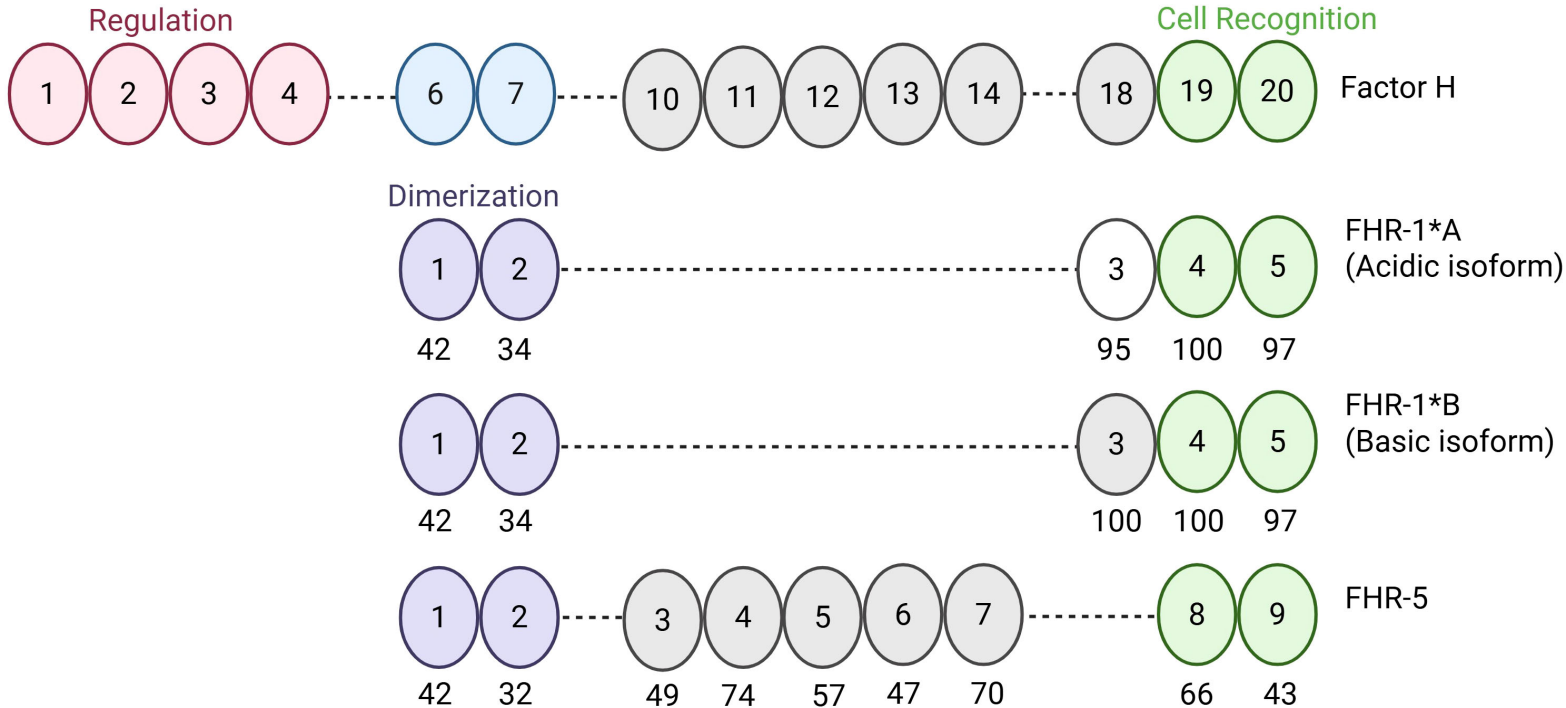
Schematic of Factor H, Factor H-related 1*A, Factor H-related 1*B, and Factor H-related 5. Factor H (FH) is composed of 20 SCRs. SCRs 1–4 mediate regulation of the alternative pathway and SCRs 19–20 mediate cell surface recognition and binding. SCRs of Factor H-related 1*A (FHR-1*A), Factor H-related 1*B (FHR-1*B), and Factor H-related 5 (FHR-5) are aligned with homologous SCRs of FH to show differences at the amino acid level. Numbers under the ovals represent percent similarity to FH. Created in BioRender. Heiderscheit, A. (2025) https://BioRender.com/s92y080.

**Figure 2:**
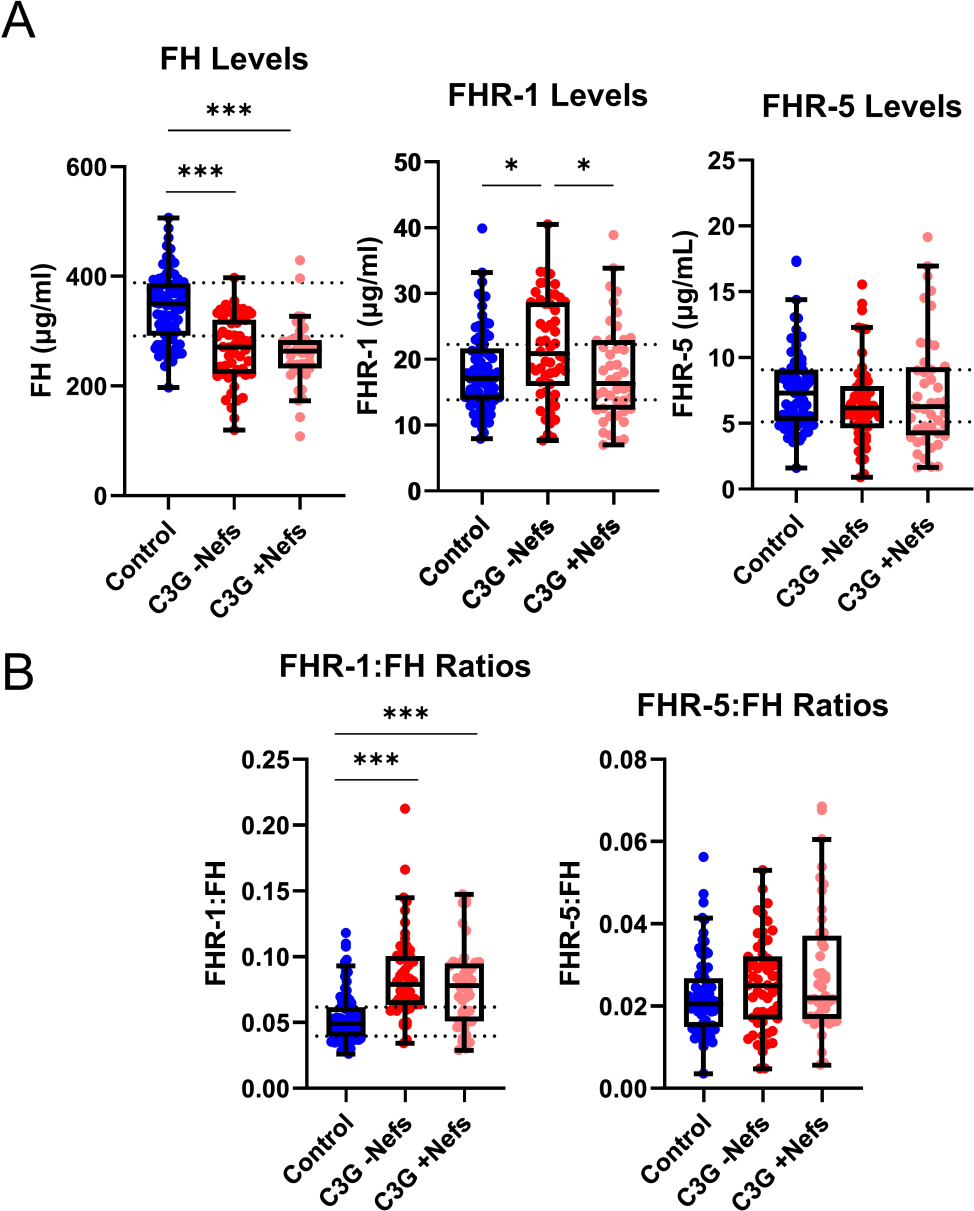
Levels and ratios of Factor H, Factor H-related 1, and Factor H-related 5 in C3 glomerulopathy. **(A)** Factor H (FH) levels are significantly decreased in all C3 glomerulopathy (C3G) cohorts while Factor H-related 1 (FHR-1) levels are significantly increased in C3G patients without nephritic factors (-Nefs) as compared to matched controls. Factor H-related 5 (FHR-5) levels are not significantly different. **(B)** The ratio of FHR-1:FH is significantly increased in C3G cohorts compared to matched controls, while FHR-5:FH ratios are not significantly different. (Kruskal Wallis test followed by Dunn’s multiple comparison test; *P < 0.05 and ***P < 0.001; Controls n = 81, C3G -Nefs n = 54, C3G +Nefs n = 44).

ELISAs were used to measure circulating levels of FH, FHR-1, and FHR-5 (**Figure 1**). C3G patients with wild-type *CFHR3-CFHR5* were stratified into two cohorts – those without nephritic factors (-Nefs) and those with nephritic factors (+Nefs), to identify any differences between patients with known drivers of disease (+Nefs) and unknown drivers of disease (-Nefs). As confounders, age and sex were also considered, but no significant differences were observed (**Supplementary Figures 2**, **3**). As shown in **Figure 1**, FH levels were significantly decreased in both patient cohorts as compared to controls (*P* < 0.001 for both)(C3G -Nefs: median 270.5μg/ml, interquartile range [IQR] 222.0-321.0; C3G +Nefs: median 263.5μg/ml, IQR 232.0-283.8; controls: median 348.6μg/ml, IQR 291.0-388.1). There were no significant differences in FH levels between C3G cohorts (**Figure 2A**). A comparison of FHR-1 levels found an increase in C3G -Nefs patients compared to both to C3G +Nefs patients and controls (*P* < 0.05 for both)(C3G -Nefs: median 20.85μg/ml, IQR 15.97-28.69; C3G +Nefs: median 17.2μg/ml, IQR 12.37-22.94; controls: median 17.20μg/ml, IQR 13.84-22.25). No significant differences were observed in FHR-5 levels (**Figure 2A**).

To determine the balance between FH and FHR-1 or FHR-5 in the circulation, we calculated relative ratios of FHR-1:FH and FHR-5:FH. FHR-1:FH ratios were significantly increased in C3G patients regardless of whether nephritic factors were present or absent as compared to controls (*P* < 0.001 for both)(C3G -Nefs: median 0.079, IQR 0.064-0.101; C3G +Nefs: median 0.0782, IQR 0.051-0.095; controls: median 0.049, IQR 0.040-0.062)(**Figure 2B**, **Supplementary Figure 4**). No significant differences were observed in FHR-5:FH ratios (**Figure 2B**). In aggregate, these data show that FH levels are decreased and FHR-1:FH ratios are increased in C3G patients.

### 3.3 Changes in FHR-1 and FH reflect chronic kidney disease

**Figure 3:**
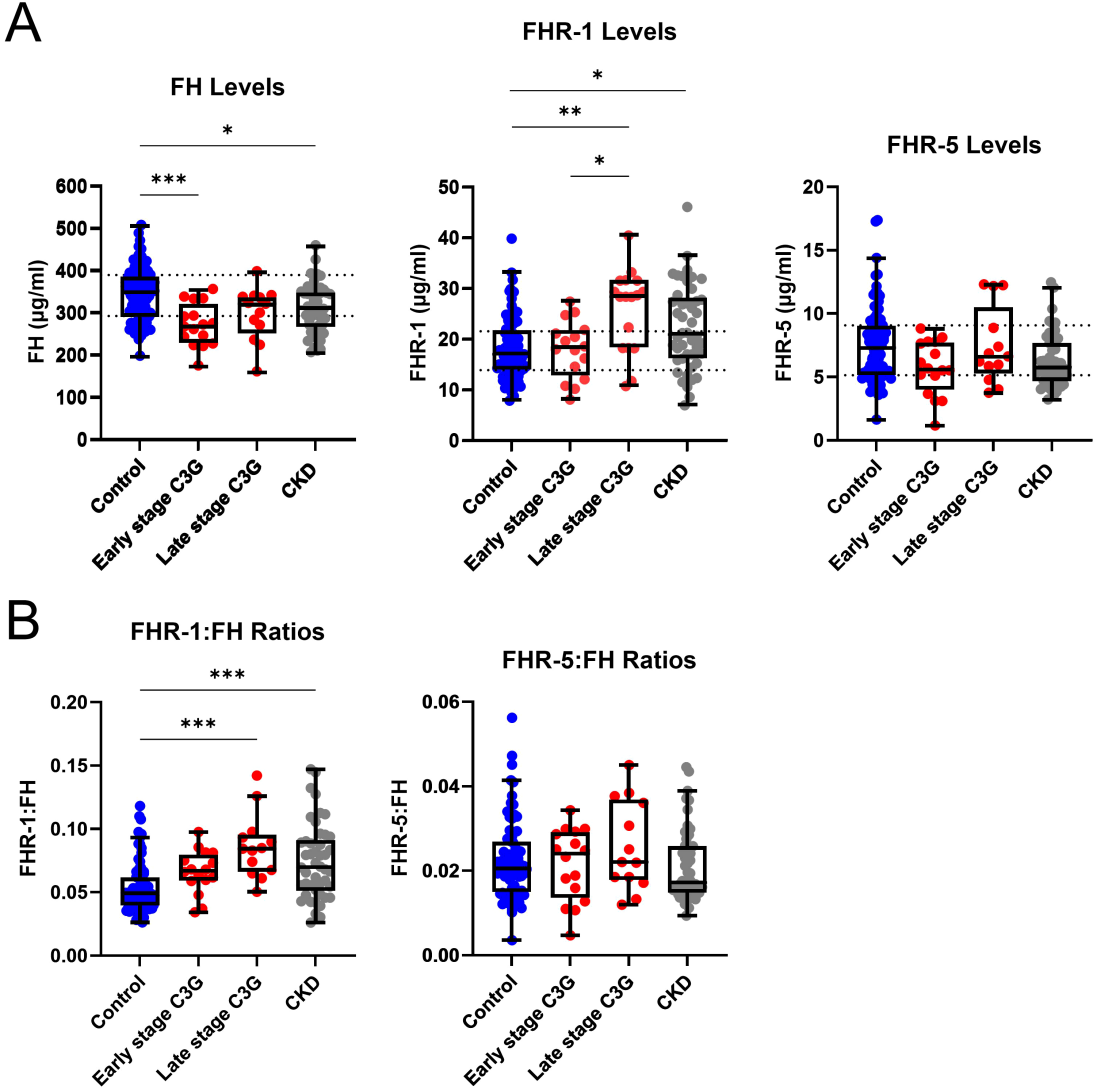
Complement protein levels and ratios by chronic kidney disease stage. **(A)** Factor H (FH) levels are significantly decreased in early stage C3 glomerulopathy (C3G) and chronic kidney disease (CKD), while Factor H-related 1 (FHR-1) levels are significantly increased in late stage C3G and CKD as compared to matched controls. Factor H-related 5 (FHR-5) levels are not significantly different. **(B)** FHR-1:FH ratios are significantly increased in late stage C3G and CKD as compared to matched controls. FHR-5:FH ratios are not significantly different. (Kruskal Wallis test followed by Dunn’s multiple comparison test; *P < 0.05, **P < 0.01, and ***P < 0.001; controls n = 81, early stage C3G n = 16, late stage C3G n = 13, CKD stage 3–4 n = 50).

To determine whether changes in FH and FHR-1 levels are specific to C3G patients or generalizable across CKD, we completed a similar study on 50 patients with CKD stages 3–4 from a variety of other causes and compared results to C3G -Nef patients in CKD stages 1-2 (early) and CKD stages 3-4 (late). Of the 54 -Nef patients, 25 patients were included based on available phenotyping data. FH levels were significantly decreased in early stage C3G and CKD patients as compared to controls (*P* < 0.001 and < 0.05, respectively)(early stage C3G cohort: median 268.5μg/ml, IQR 230.0-322.0; CKD cohort: 312.8μg/ml, IQR 267.8-349.7; controls: median 348.6μg/ml, IQR 291.0-388.1). In late stage C3G, FH levels were not significantly different from controls, though a downward trend was observed. Furthermore, FH levels in both the late stage C3G and CKD cohorts were not significantly different as compared to each other (**Figure 3A**). FHR-1 levels were significantly increased in late stage C3G as compared to early stage C3G and controls (*P* < 0.01 and < 0.05, respectively)(late stage C3G: median 28.44μg/ml, IQR 18.32-31.59; early stage C3G: 18.36μg/ml, IQR 12.77-21.73; controls: median 17.20μg/ml, IQR 13.84-22.25), as well as in CKD as compared to controls (*P* < 0.05)(CKD: median 20.98μg/ml, IQR 16.14-28.10; controls: median16.94μg/ml, IQR 13.82-21.49). No significant differences were observed in FHR-5 levels (**Figure 3A**).

FHR-1:FH ratios in late stage C3G and CKD were also significantly increased as compared to controls (*P* < 0.001 for both)(late stage C3G: median 0.084, IQR 0.066-0.095; CKD: median 0.070, IQR 0.051-0.091; controls: median 0.049, IQR 0.040-0.062), but there were no differences between the C3G and CKD cohorts. No significant differences were found for FHR-5:FH ratios (**Figure 3B**). Collectively, these results mean that as kidney function declines, FHR-1:FH ratios increase regardless of the underlying cause of renal disease.

### 3.4 FHR-1 and FHR-5 increase C3b deposition on cell surfaces

**Figure 4:**
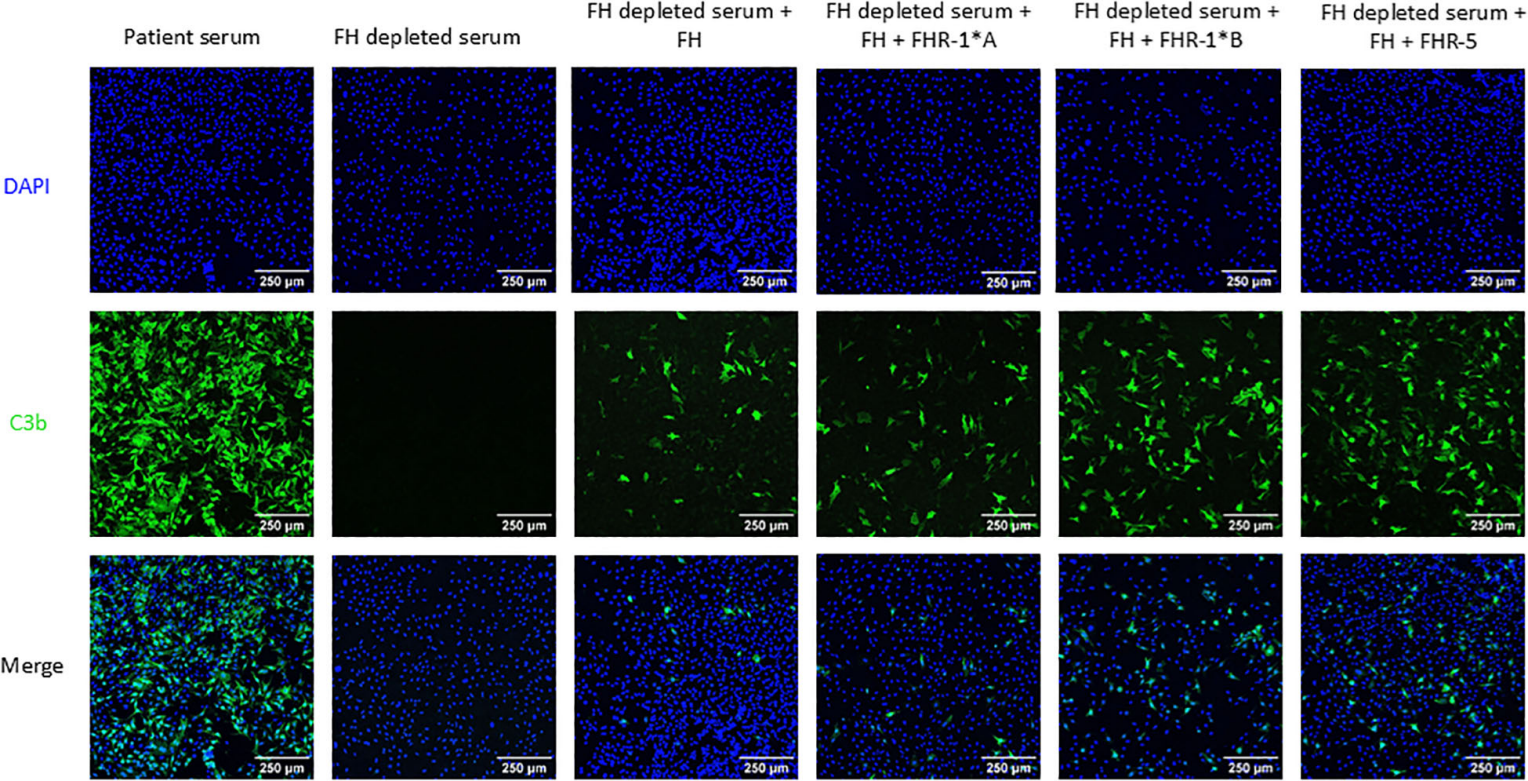
C3b deposition on mouse mesangial cells. Mouse mesangial cells (MES-13) were incubated with patient serum or Factor H (FH) depleted serum with or without human FH and human Factor H-related 1*A (FHR-1*A), human Factor H-related 1*B (FHR-1*B), and/or human Factor H-related 5 (FHR-5). Cells were then stained for C3b deposition (green) and nuclei (blue). All three FHR proteins significantly increased C3b deposition as compared to FH alone, with odds increase in deposition for FHR-1*A, FHR-1*B and FHR-5 of 2.9, 11.6, and 7.3, respectively, based on 10 experimental replicates. 250μm scale bar is shown. (Logistic regression model; *P* < 0.001 for all).

C3b deposition was assessed as a measure of local complement activity in human FH-depleted serum supplemented with FH, rFHR-1*A, rFHR-1*B and/or rFHR-5 on cultured MES-13 cells (**Figure 1**). A CKD patient with a pathogenic variant in *G6PD* (rs1050829) leading to high levels of complement activation was used as a positive control. C3b visualization was not possible in FH-depleted serum due to uncontrolled consumption of C3. Therefore, we added FH back into the FH-depleted serum at various concentrations to identify the point at which C3b deposition was moderate, thereby allowing for the visualization of changes in deposition under other experimental conditions (**Supplementary Figures 5**-**7**). FH-depleted serum supplemented with FH and all FHRs conditions significantly increased C3b cell surface deposition with odds increase in deposition for rFHR-1*A, rFHR-1*B, and rFHR-5 of 2.9, 11.6, and 7.3, respectively (*P* < 0.001 for all)(**Figure 4**). These results are further supported by an FHR-5 dose-dependent increase in C3b deposition (**Supplementary Figure 8**). Combined, these results show that FHR-1 and FHR-5 enhance C3b deposition on MES-13 cell surfaces.

### 3.5 Heparan sulfate cleavage increases C3b deposition on cell surfaces

**Figure 5:**
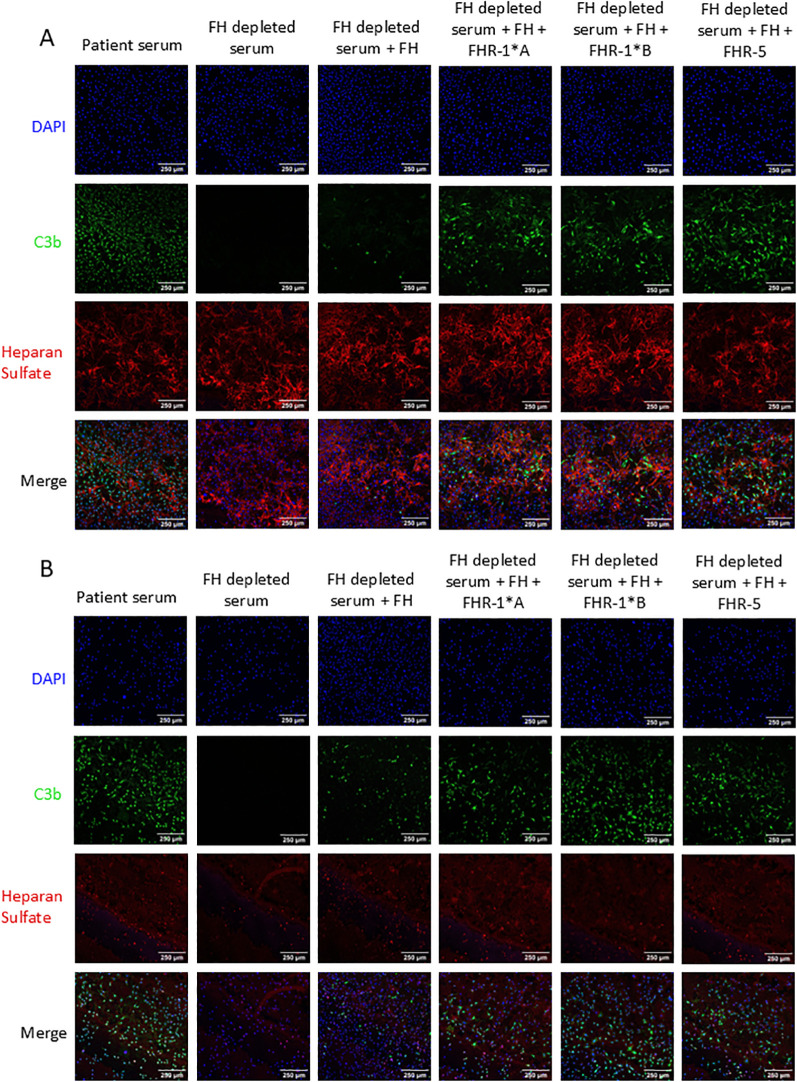
C3b deposition on mouse mesangial cells pretreated with heparinase. Mouse mesangial cells (MES-13) were treated with **(A)** media or **(B)** heparinase followed by incubation with FH-depleted serum supplemented with C3 and FH or additionally FHR-1*A, FHR-1*B or FHR-5. Cells were stained for C3b deposition (green), heparan sulfate (red), and nuclei (blue). C3b deposition was significantly increased by heparinase treatment with odds increase in deposition for FH, FHR-1*A, FHR-1*B, and FHR-5 of 2.9, 4.7, 5.5, and 1.8, respectively, based on 5 experimental replicates. 250μm scale bar is shown. (Logistic regression model; *P* < 0.001 for all).

Heparan sulfate (HS) is a key component of the glycocalyx and plays a role in FH and potentially FHR-1 and FHR-5 binding. Thus, to assess the impact of HS alterations on C3b deposition, we treated MES-13 cells with heparinase to cleave α(1→4) glycosidic bonds in HS chains (**Supplementary Figure 9**). C3b deposition was significantly increased on all heparinase treated cells as compared to non-treated cells with odds increase in C3b deposition for FH, rFHR1*A, rFHR1*B, and rFHR5 of 2.9, 4.7, 5.5, and 1.8, respectively (P < 0.001 for all) (**Figure 5**). In aggregate, these results mean that HS exposure to heparinase increases C3b deposition on MES-13 cell surfaces.

### 3.6 FHR-1 and FHR-5 bind to low and non-sulfated heparan sulfate glycans

**Figure 6:**
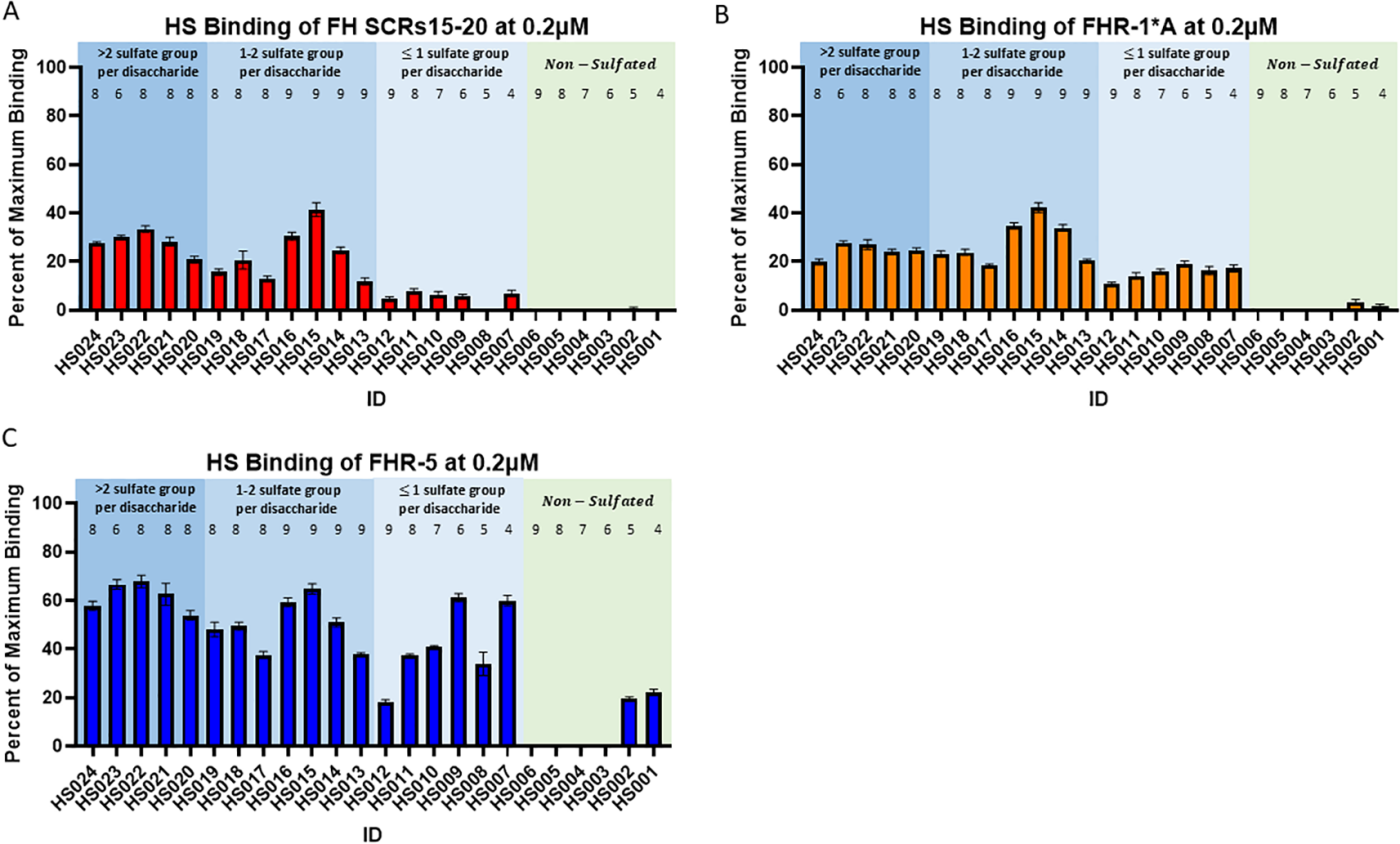
Binding of Factor H, Factor H-related 1*A, and Factor H-related 5 to heparan sulfate glycans. Plots showing the average percent of maximum binding of 6 replicates for **(A)** Factor H (FH) SCRs15-20, **(B)** Factor H-related 1*A (FHR1*A), and **(C)** Factor H-related 5 (FHR5) to various heparan sulfate (HS) glycans. Numbers above bars represent sugar residues per disaccharide. Standard error of the mean is shown.

A HS microarray was used to examine binding of human FH SCRs 15-20, rFHR-1*A, and rFHR-5. Strong binding to HS glycans with 8–9 sugar residues and greater than one sulfate group per disaccharide was observed with all three proteins, however binding decreased in all cases when sulfation dropped below one group per disaccharide (**Figure 6**). Only rFHR-1*A and rFHR5 bound to non-sulfated HS glycans (**Figures 6B, C**). These results indicate that while FH SCRs 15-20, rFHR-1*A, and rFHR-5 are all capable of binding to long chain, highly sulfated HS structures, only rFHR-1*A and rFHR-5 have a high binding affinity for low and non-sulfated, short-chain HS.

## 4 Discussion

In this study we explored the interrelationship between FHR-1 and FHR-5 with FH in C3G patients. Here, we confirm previous studies showing that deletion of *CFHR3-CFHR1* is less frequent in C3G patients. This observation prompted us to compare circulating levels of FHR-1, FHR-5, and FH in C3G patients and controls (all with two copies of *CFHR3-CFHR5*). We found that FHR-1:FH ratios were increased in C3G patients relative to controls, but that this increase was not disease specific. Rather, it was a hallmark of poor kidney function and was present in a second cohort of patients with CKD due to a variety of causes (**Supplementary Table 1**). While the functional spectrum of FHR-1 and FHR-5 remains to be definitively defined, our *in vitro* assays showed that FHR-1 and FHR-5 compete with FH to increase C3b deposition on MES-13 cell surfaces and that deposition is enhanced by HS cleavage. These observations suggest that in the setting of reduced kidney function, complement activity is increased.

**Figure 7:**
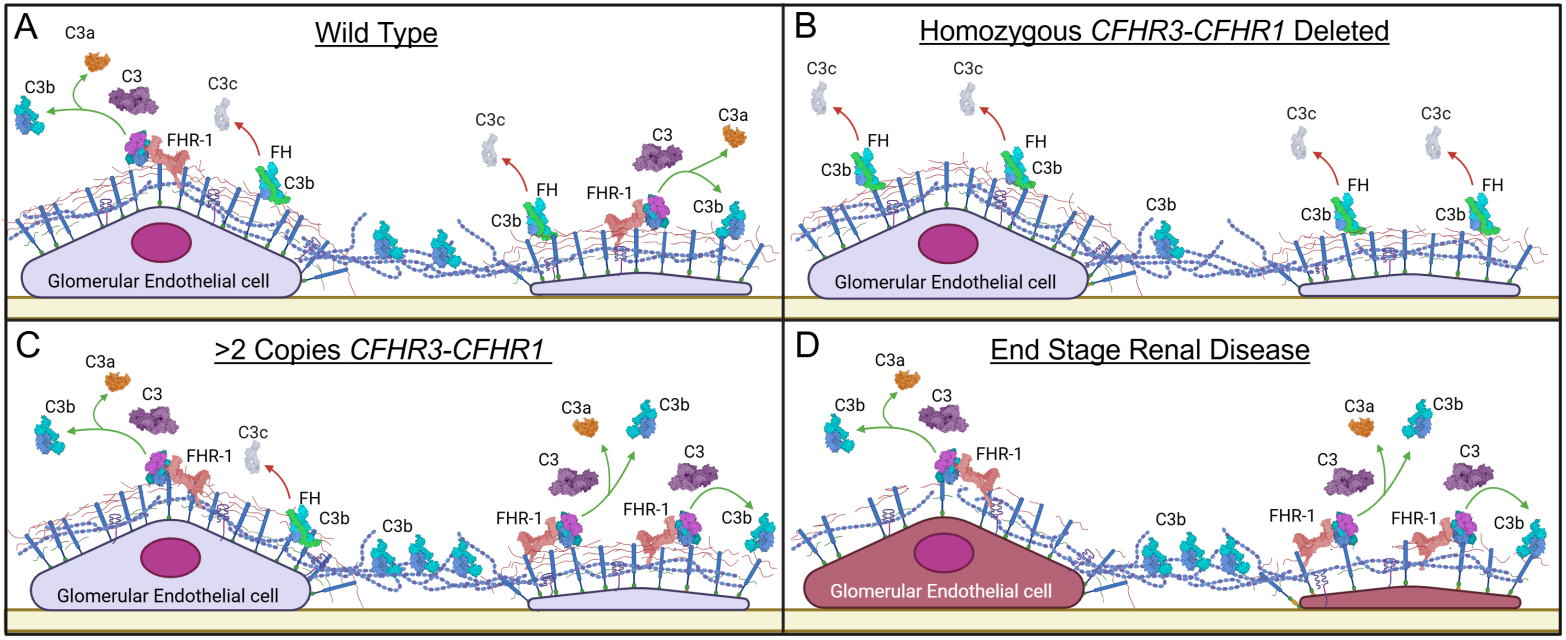
Proposed working model for the pathogenic mechanism of Factor H-related 1. **(A)** Factor H (FH) functionally competes with Factor H-related 1 (FHR-1) for binding to cell surfaces, generating a balance between alternative pathway (AP) regulation and progression. **(B)** In homozygous deleted *CFHR1* individuals, FH has less competition for binding to the glycocalyx (due to deletion of FHR-1); increased binding of FH provides more effective AP regulation. **(C)** In individuals with increased *CFHR1* copy numbers, the increased FHR-1 outcompetes FH for binding, decreasing AP regulation and shifting the balance in favor of AP progression. **(D)** In end stage renal disease, the increase in FHR-1 and decrease in FH favor C3b deposition and complement activity. Additionally, damage to the glycomatrix also impacts differential binding of FH and FHR-1, further enhancing complement activity. Created in BioRender. Heiderscheit, A. (2025) https://BioRender.com/s92y080.

Variation in *CFHR1* copy numbers is the consequence of exceptionally high homology over the *CFH-CFHR5* genomic region, which promotes large segmental deletions and duplications (**Supplementary Figure 10**). The most common of these CNVs is the *CFHR3-CFHR1* deletion, with homozygous deletion of both copies of the *CFHR3-CFHR1* genes present in 1.8% of NFE and 8.5% of AFR (gnomad.broadinstitute.org). The protective effect of the *CFHR3-CFHR1* deletion has been previously described in two Spanish C3G cohorts, immunoglobulin A nephropathy (IgAN), and age-related macular degeneration (20, 40–45). The association of protection with the absence of *CFHR3-CFHR1* is due to enhanced local complement regulation; greater than two copies of *CFHR3-CFHR1* increase circulating FHR-1, decrease FH regulation, and explain how copy numbers of *CFHR3-CFHR1* act as a pathogenic factor in development of C3G and potentially other complement-mediated diseases (**Figure 7**).

The yin-and-yang relationship between FHR-1 and FH suggests an important role for these proteins in controlling complement activity in specific microenvironments (16, 17, 29, 39, 46–48). Indirect evidence of high spectral counts of FHR-1 and FHR-5 in the dense deposits extracted from C3G kidney biopsies in addition to the association of the *CFHR3-CFHR1* deletion with better renal outcomes in C3G prompted us to compare circulating levels of FHR-1, FHR-5, and FH in C3G patients and controls with two copies of *CFHR3-CFHR5* (**Supplementary Figure 11**) (20, 33). Our findings show that FHR-1:FH ratios are increased in C3G patients but that this increase is not disease specific. Rather it reflects poor kidney function as it was seen in a second cohort of patients with CKD due to a variety of causes (**Supplementary Table S1**). FHR-1 studies conducted on immunoglobulin A nephropathy and autosomal dominant polycystic kidney disease have reported a similar trend, in addition to a negative correlation between FHR-1 levels and estimated glomerular filtration rate (eGFR) (39, 47, 49). We did not observe any differences in FHR-5 levels or ratios, in contrast to a report of decreased levels in a cohort of immune complex-mediated membranoproliferative glomerulonephritis/C3G patients (50). This discrepancy could reflect differences in patient cohorts. Currently we do not know why there is a rise in FHR-1:FH ratios as CKD progresses. Several studies have shown cytokines such as interlulin-1, interferon-γ, and tumor necrosis factor-α can regulate gene expression of complement components in glomerular cell lines. Thus, one possible mechanism behind alterations in circulating protein levels could be transcriptional variance driven by local or systemic inflammatory cytokine production during renal injury (51–53). Regardless, in the setting of CKD, FHR-1:FH ratios rise, and this rise impacts complement activity.

HS is one of the key components of the glycocalyx to which FH binds to regulate complement activity (54). Alterations in HS chain length and sulfation patterns impact this binding and therefore impact FH-mediated complement regulation (55, 56). A recent study has shown that FHR-1 and FHR-5 also bind to HS but with different ligand specificity as compared to FH. FHR-1 and FHR-5 preferentially bind to de-sulfated HS derivatives, which are a proxy for heparanase-induced damage to HS (25). Importantly, the distribution of HS in the CKD kidneys, including C3G, has been quantified and found to decrease while heparanase increases (57–61). To model this condition and assess the impact of altered HS on FHR-1, FHR-5, and FH binding to cell surfaces, we quantified C3b deposition on MES-13 cells with and without heparinase treatment. Our non-treatment data are in accordance with previous findings that demonstrate FHR-1 and FHR-5 interfere with FH-mediated complement regulation, while our treatment data show that HS cleavage further enhances C3b deposition (29, 47). These finding are supported by our microarray data, which show that FHR-1 and FHR-5 can bind to low and non-sulfated, short chain HS while FH cannot (62, 63). Overall, these results suggest that alteration to HS in the setting of CKD changes the binding of FH and FHRs to the glycocalyx, which potentially contributes to a labile complement cascade (**Figure 7**).

Identification of FHR-1 as an enhancer of C3b deposition provides a novel potential mechanism for progression of kidney disease in CKD patients. Specifically, we show that in a variety of CKDs, including C3G, diabetic nephropathy and autosomal dominant polycystic kidney disease, there is a rise in FHR-1:FH ratios in later stages of disease (**Supplementary Table 1**). This shift enhances FHR-1 activity, which contributes to increased C3b deposition on cell surfaces, possibly further compounding renal damage by activating complement. (**Supplementary Figure 12**). These finding also suggest that in the cohort of C3G patients with no known drivers of disease (i.e. negative for nephritic factors and genetic mutations in complement genes), local complement dysregulation in the glomerular microenvironment may be driving disease.

While this study provides valuable insight to the complexity of complement activity in the glomerular microenvironment, several limitations may impact its generalizability. Firstly, in testing C3b deposition, only select complement proteins were used, a constraint that may have impacted the dynamics of C3b generation and deposition and inadequately represent the overall impact of FHR-1 and FHR-5. Secondly, to study binding to cell surfaces we used a mouse cell line as opposed to a human cell line. Species-specific differences in complement cell surface regulators could result in different C3b deposition patterns between mice and humans, thus additional studies using human cell lines should be undertaken (64, 65). Thirdly, to induce HS damage on cell surfaces we focused on one type of insult, heparinase. In a physiological setting, kidney injury is far more complex. Reactive oxygen species, for example, are also released and concurrently damage the structure of HS (66–69). Thus, follow-up studies assessing the impact of different drivers of kidney injury on complement activity are necessary.

In summary, our study shows that in the setting of CKD, there is a rise in FHR-1:FH ratios, which together with damaged HS architecture contribute to increased complement activity. If confirmatory studies demonstrate that complement-mediated damage enhances progression of kidney injury in CKD patients, anti-complement therapy may play a role in altering disease progression.

## Data Availability

The raw data supporting the conclusions of this article will be made available by the authors, without undue reservation.
