# Supplementary material for: Factor H-related 1 and heparan sulfate architecture contribute to complement dysregulation in C3 glomerulopathy
Source: Front Immunol. 2025 May 16;16:1589674. doi: 10.3389/fimmu.2025.1589674 (PMC12122740; doi:10.3389/fimmu.2025.1589674)
Supplement: Supplementary file 1 [file DataSheet1.docx]

**Supplementary Materials**


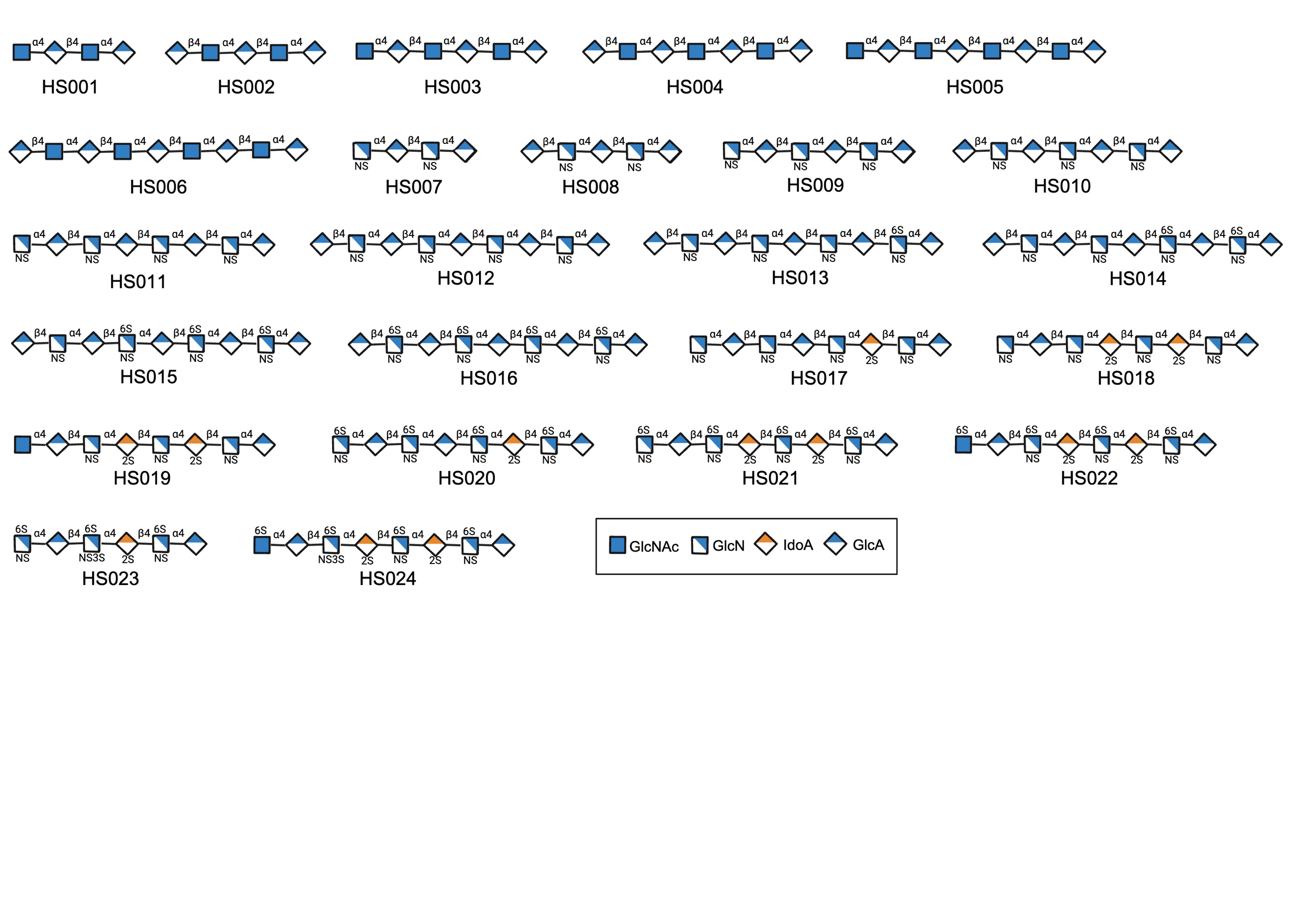


**Supplemental Figure 1.** Heparan sulfate (HS) glycans used in the microarray analysis. (*N-*acetyl-D-glucosamine, GlcNAc; D-glucosamine, GlcN; L-iduronic acid, IdoA; D-glucuronic acid, GlcA). Created in BioRender. Heiderscheit, A. (2025) https://BioRender.com/h08e848


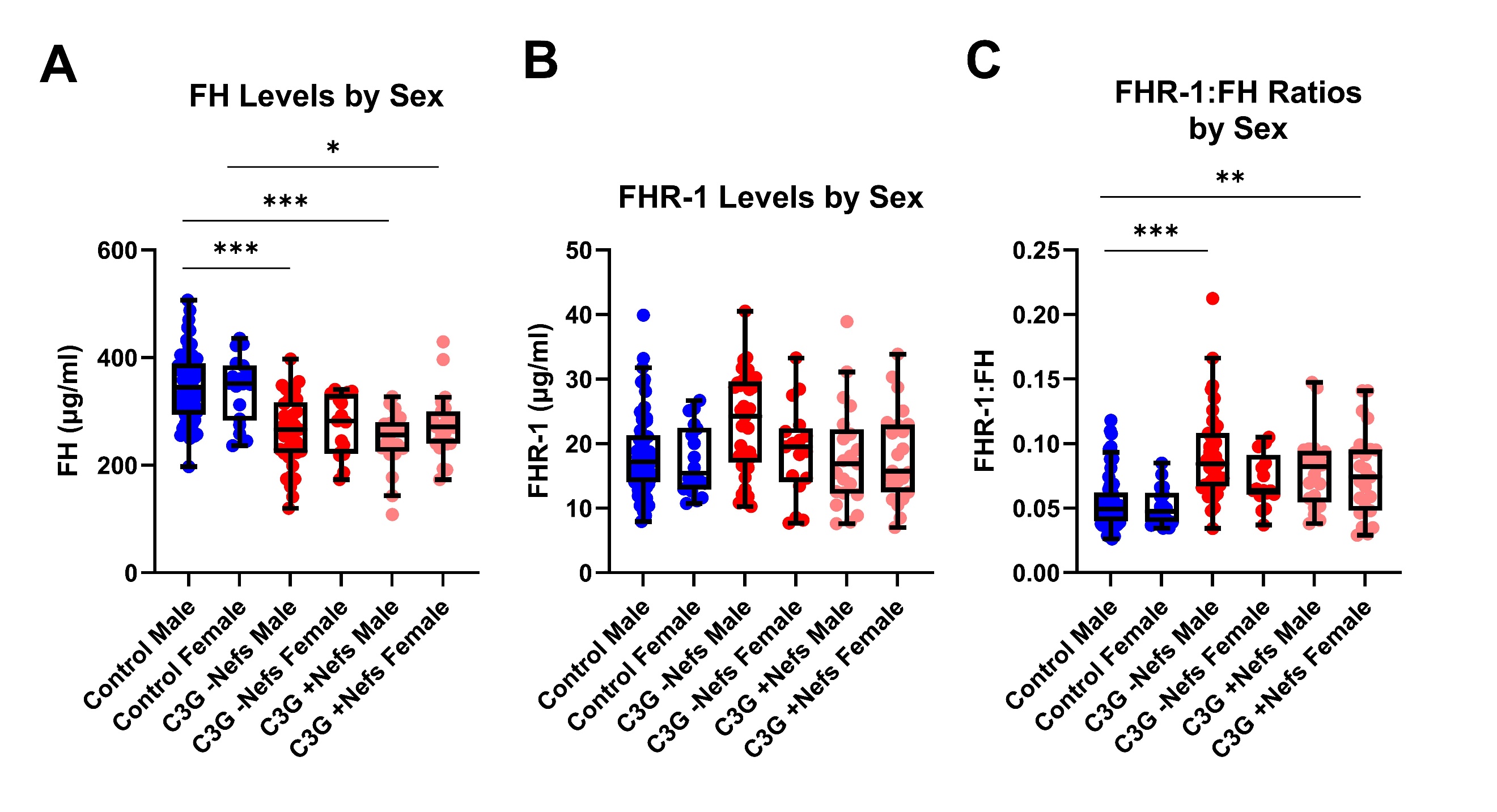


**Supplemental Figure 2. Factor H-related 1 levels and ratios in C3 glomerulopathy by sex.** No significant sex differences were observed for **(A)** Factor H (FH) levels, **(B)** Factor H-related 1 (FHR-1) levels, or **(C)** FHR-1:FH ratios. (Kruskal-Wallis test followed by Dunn’s multiple comparison test; **P* < 0.05, ***P* < 0.01, and ****P* < 0.001; Control Male n = 63, Control Female n = 18, C3G -Nefs Male n = 37, C3G -Nefs Female n = 17, C3G +Nefs Male n = 21, C3G +Nefs Female n = 23)


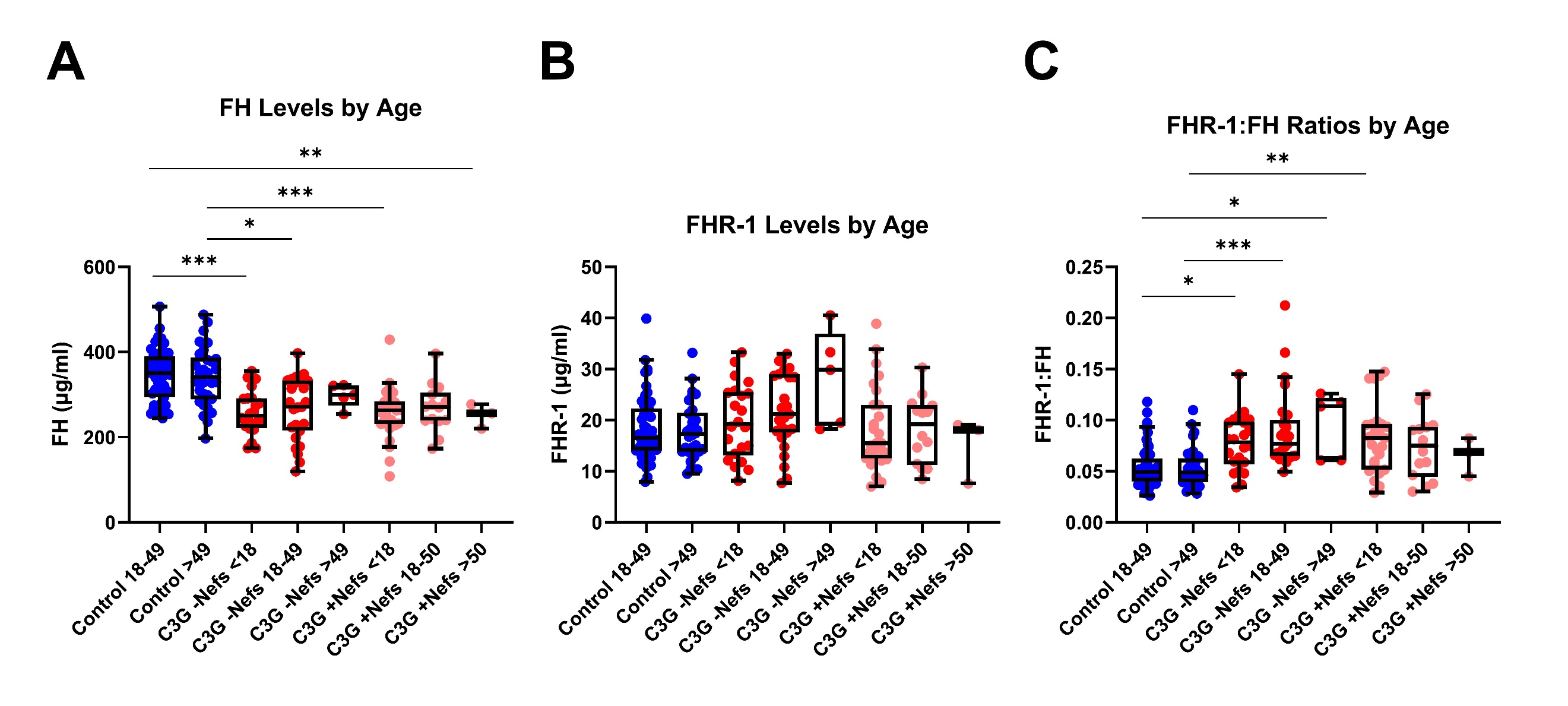


**Supplemental Figure 3. Factor H-related 1 levels and ratios in C3 glomerulopathy by age.** No significant age differences were observed for **(A)** Factor H (FH) levels, **(B)** Factor H-related 1 (FHR-1) levels, or **(C)** FHR-1:FH ratios. (Kruskal-Wallis test followed by Dunn’s multiple comparison test; **P* < 0.05, ***P* < 0.01, ****P* < 0.001, and ****P* < 0.001; Controls 18-49 n = 51, Controls >49 n = 30, C3G -Nefs <18 n = 22, C3G -Nefs 18-49 n = 27, C3G -Nefs >49 n = 5, C3G +Nefs <18 n = 27, C3G +Nefs 18-49 n = 14, C3G +Nefs >49 n = 3)


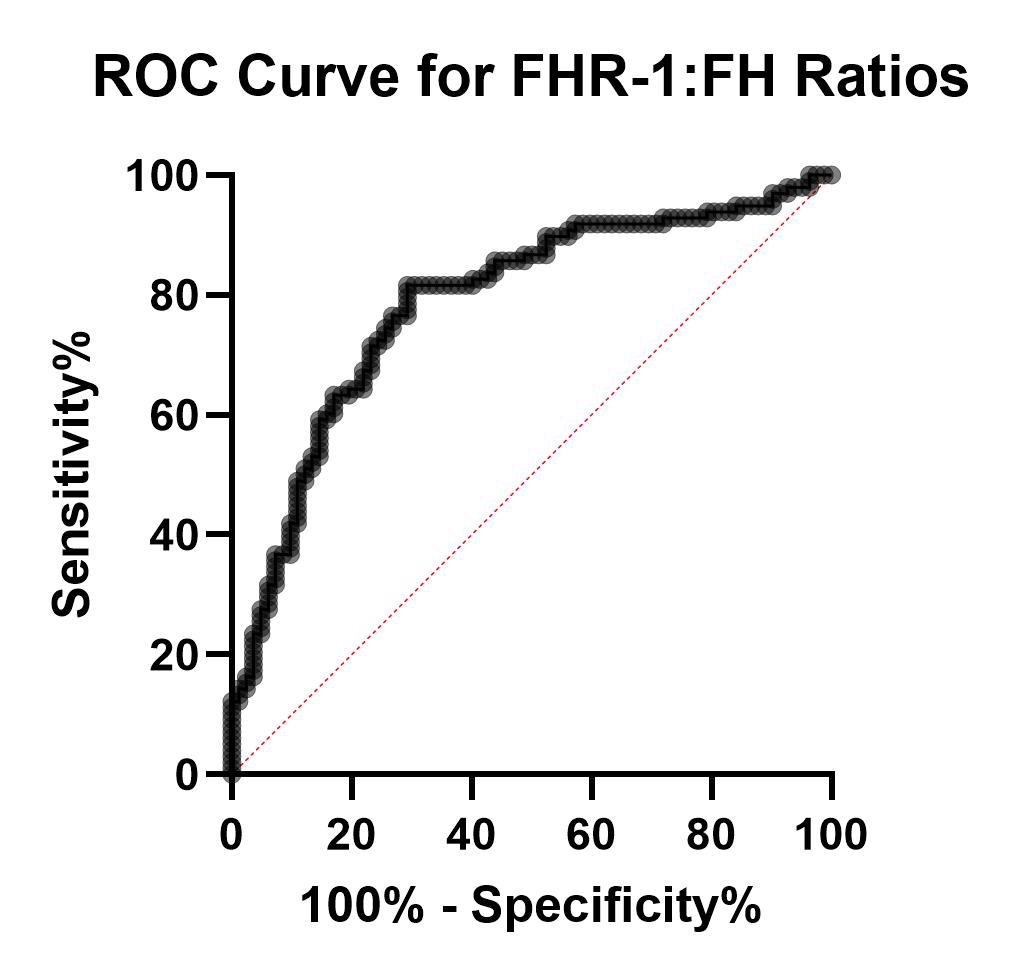


**Supplemental Figure 4. Receiver operating characteristic curve for Factor H-related 1 to Factor H ratios.** Factor H-related 1 to Factor H (FHR-1:FH) ratios for C3 glomerulopathy patients were compared to controls. Area under the curve is 0.7854; *P =* 1.79e-11; optimal threshold analysis is 0.057. (Controls n = 81, C3G n = 98)

A

B


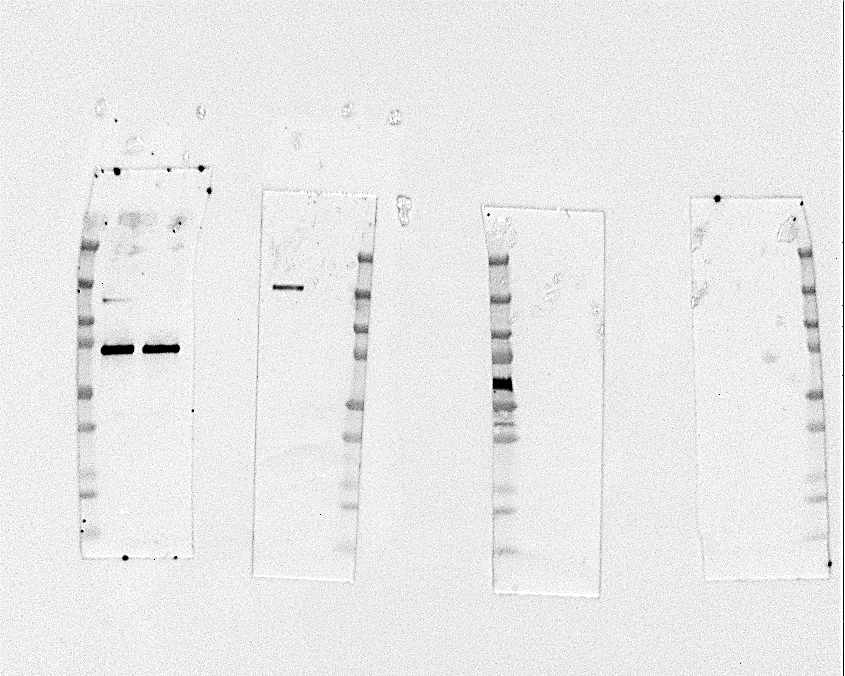


75kD

Factor I

Normal human serum

FH depleted serum


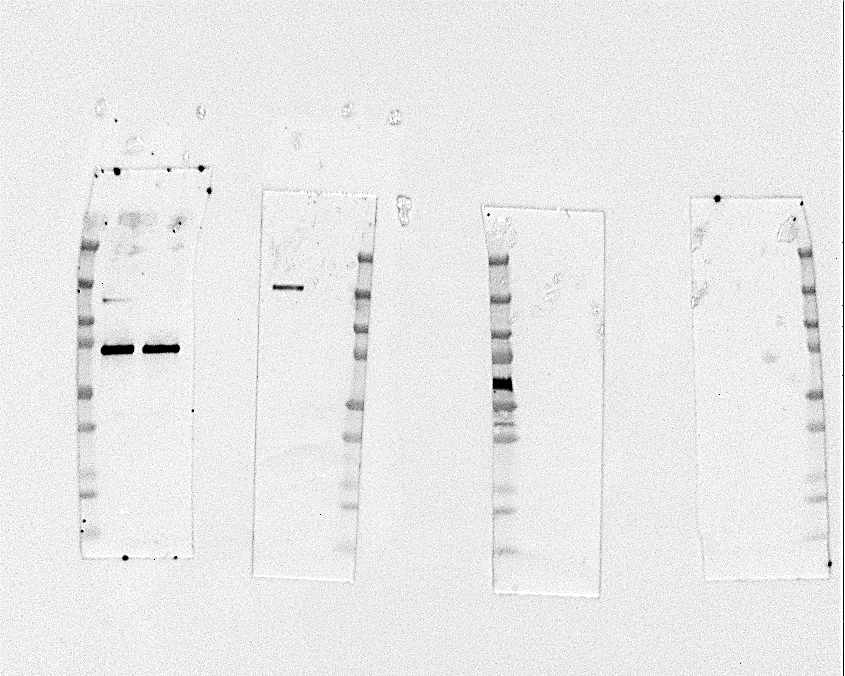


Factor H

150kD


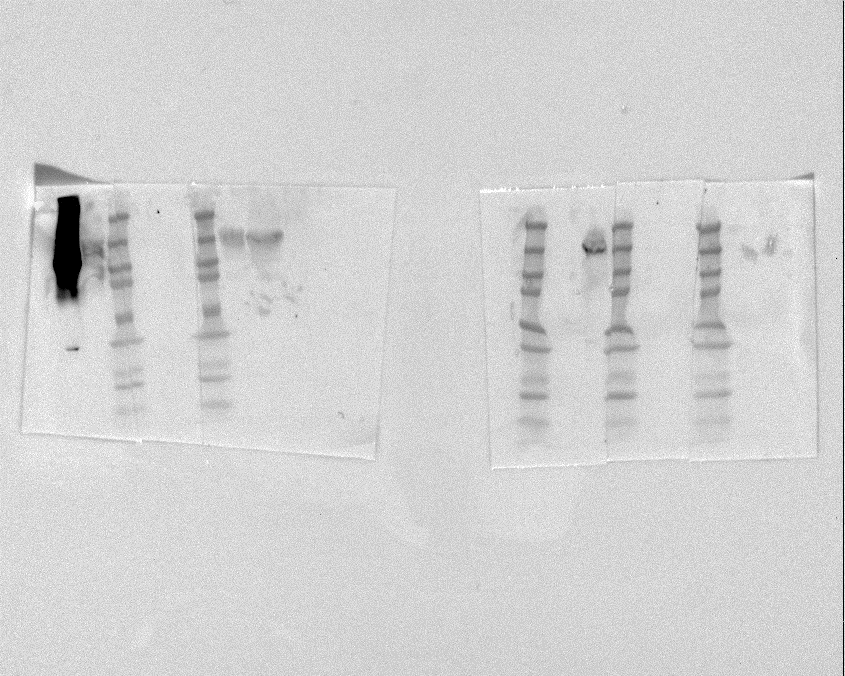


37kD

Factor H-like 1

Ladder

50kD

37kD

25kD


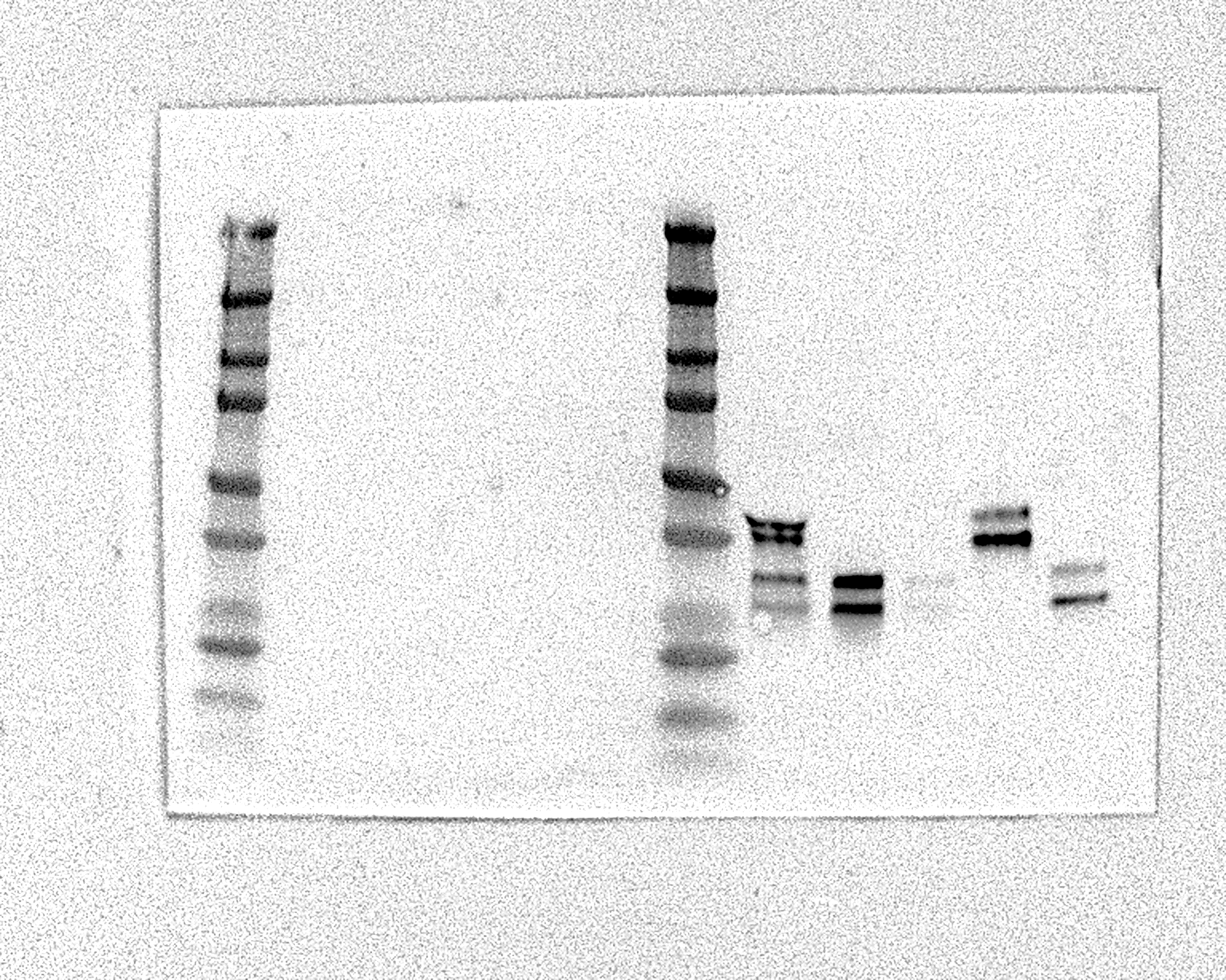


Normal human serum

*CFHR3-CFHR1 deletion*

FH depleted serum

FHR-1

FHR-2

Ladder

**Supplemental Figure 5. Characterization of Factor H (FH) depleted serum. (A)** FH depleted serum is fully depleted of FH and Factor H-like 1 but has Factor I. **(B)** FH depleted serum is also fully depleted of Factor H-related 1 (FHR-1) with trace amounts of Factor H-related 2 (FHR-2).


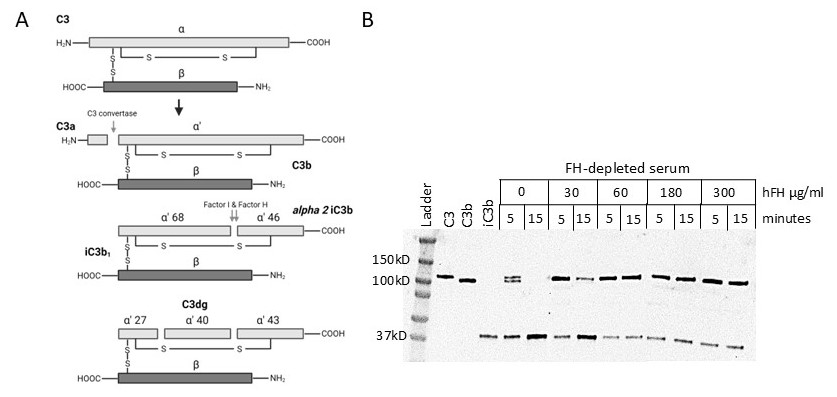


**Supplemental Figure 6. Complement activation in Factor H (FH) depleted serum.** **(A)** Fragments generated by C3 cleavage. **(B)** Time course of C3 fragment production in FH depleted serum with alternative pathway activation buffer and increasing concentrations of FH.

**
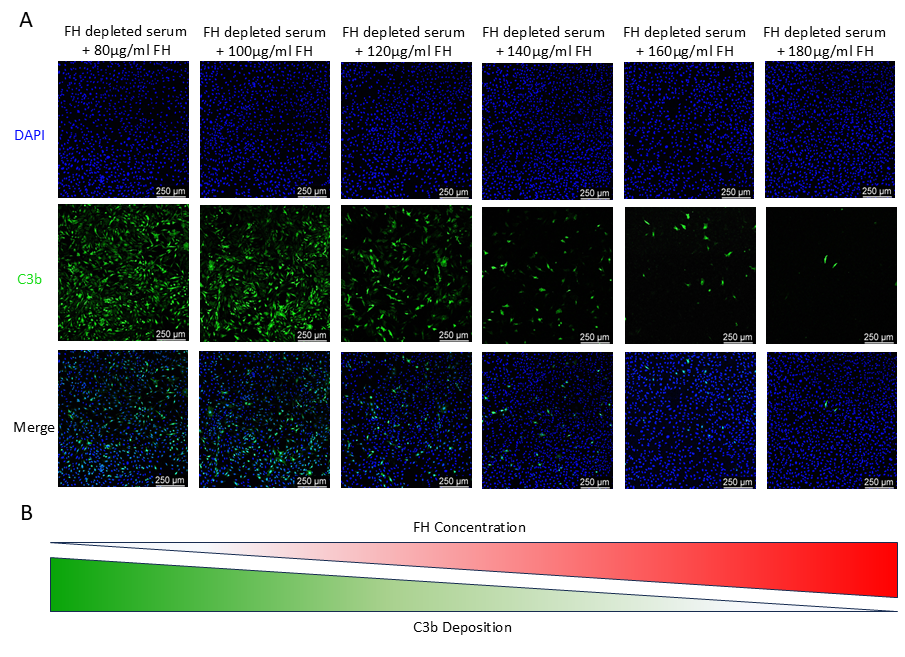
**

**Supplemental Figure 7**. **C3b deposition Factor H dose curve**. **(A)** Mouse mesangial cells (MES-13) were incubated with Factor H (FH) depleted serum supplemented with increasing concentrations of human FH - 80μg/ml, 100μg/ml, 120μg/ml, 140μg/ml, 160μg/ml and 180μg/ml. Cells were stained for C3b deposition (green) and nuclei (blue). 250μm scale bar is shown. **(B)** C3b deposition and FH concentration have an inverse relationship - as the concentration of FH increases, alternative pathway activity is regulated, decreasing C3b deposition. If the Factor H-related proteins (FHRs) regulate complement activity like FH, C3b deposition for FH plus FHRs will be similar to 180μg/ml of FH; alternatively, if the FHRs antagonize FH, C3b deposition for FH plus FHRs will be similar to the lower doses of FH.

**
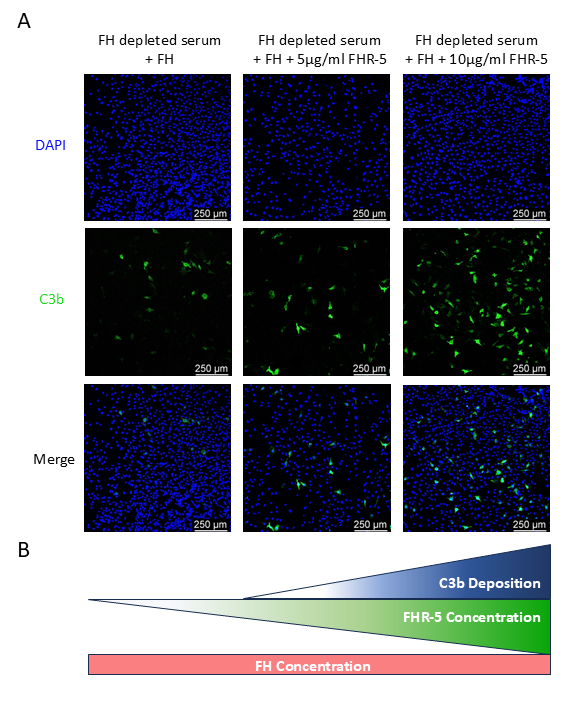
**

**Supplemental Figure 8. C3b deposition Factor H-related 5 dose curve. (A)** Mouse mesangial cells (MES-13) were incubated with Factor H (FH) depleted serum supplemented with human FH and recombinant Factor H-related 5 (FHR-5) at physiological concentrations of 5μg/ml or 10μg/ml. Cells were stained for C3b deposition (green) and nuclei (blue). 250μm scale bar is shown. **(B)** FHR-5 antagonizes FH regulation, increasing C3b deposition in a dose-dependent manner.

**
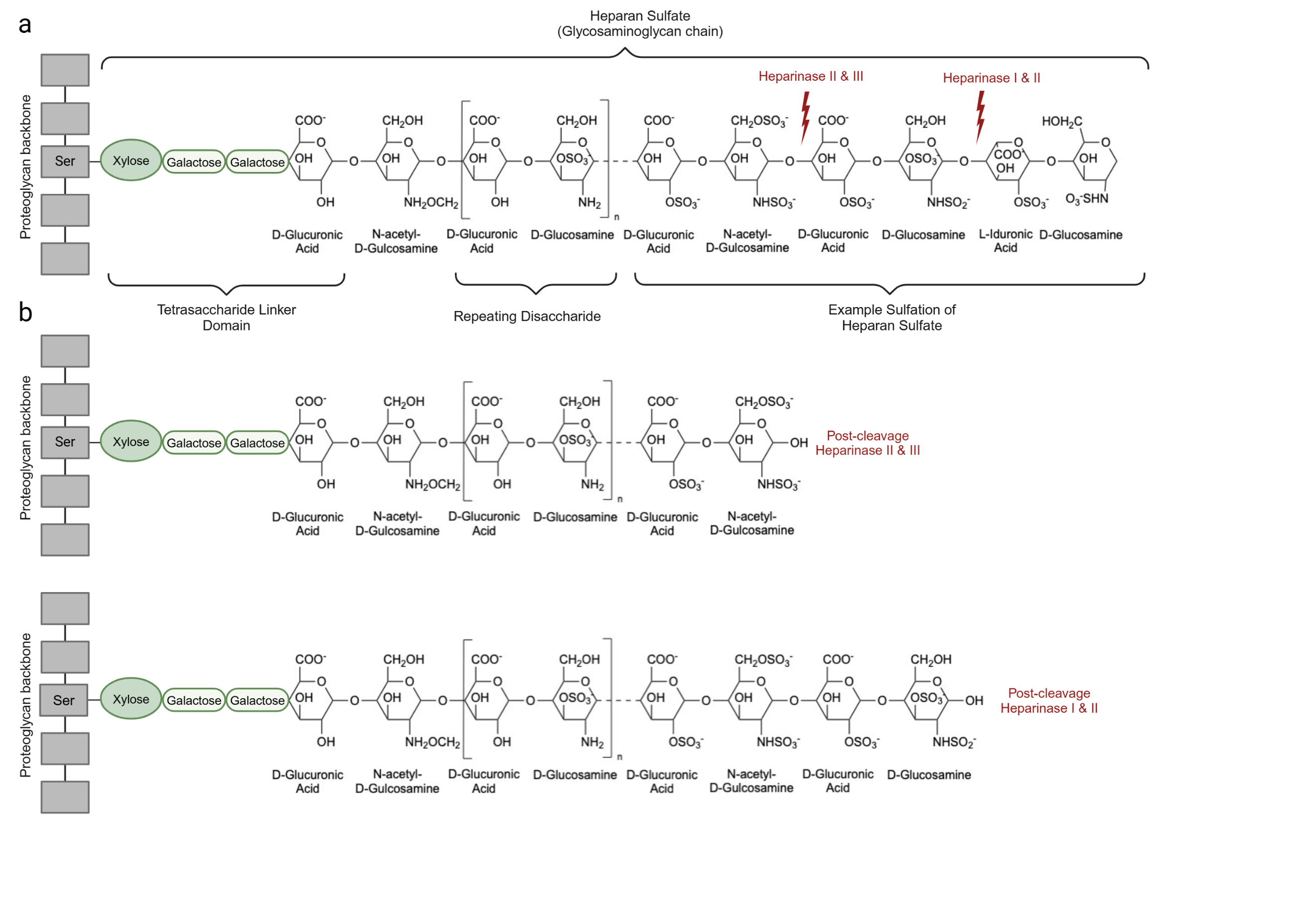
**

**Supplemental Figure 9. Heparan sulfate biosynthesis and cleavage by heparinase.**  Biosynthesis begins with the formation of a tetrasaccharide linker domain, consisting of xylose-galactose-galactose-glucuronic acid, attached to a serine in a proteoglycan backbone. Thereafter, initiation of the heparan sulfate (HS) chain formation starts with the attachment of N-acetylglucosamine followed by repeating disaccharides of glucuronic acid and glucosamine. Modification of residues are then conducted by glycosyltransferases, N-deacetylation/N-sulfotransferases, C5-epimerase, and 6-O/2-O/3-O-sulfotransferases. The structure of HS is variable; an example is shown. Heparinase II and III are ligases that catalyze alpha 1-4 eliminative reactions of glycosidic bonds between N-acetylglucosamine and glucuronic acid/iduronic acid generating truncated HS chains and oligosaccharides. Cleavage is variable, examples are shown.

**
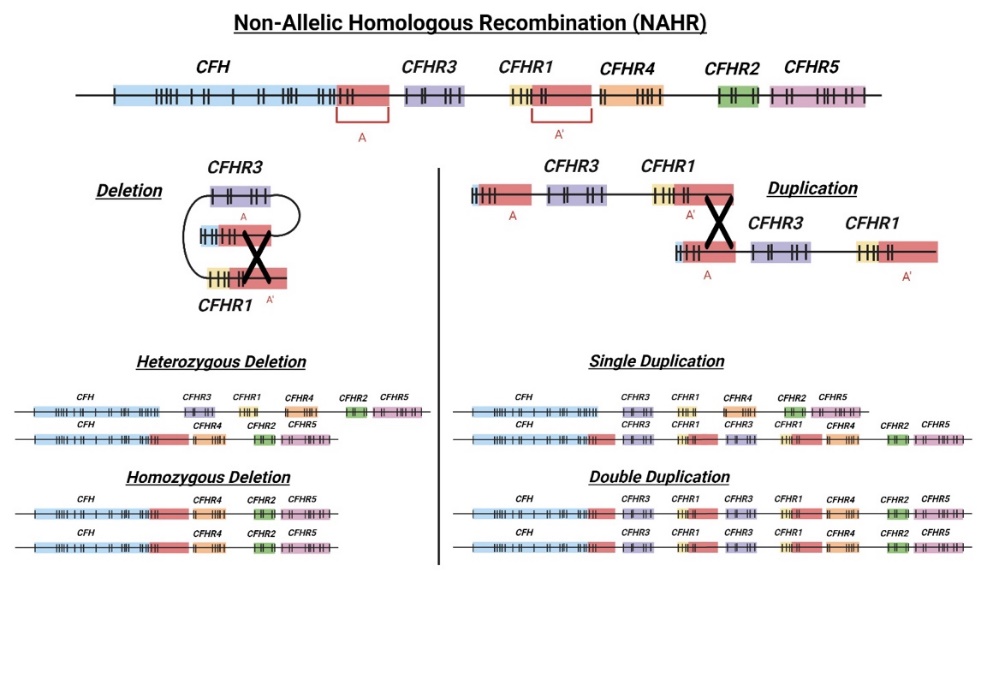
**

**Supplemental Figure 10. Non-allelic homologous recombination of *CFHR3-CFHR1.*** The *CFH* gene cluster (shown in chromosomal order) is on chromosome 1q32 in the regulators of complement activation (RCA) region. Deletions and duplication of *CFHR3-CFHR1* arise through non-allelic homologous recombination (NAHR), a process that involves the loss (or gain) of genetic material at positions of high homology (red boxes). This process is key to generation of copy number variants (CNVs) and structural variations. NAHR *CFHR3-CFHR1* deletions result in heterozygous (i.e., 1 copy of *CFHR3-CFHR1*) or homozygous deletions (i.e., no copies of *CFHR3-CFHR1*)*.* NAHR *CFHR3-CFHR1* duplications result in a gain of genetic material, generating 3 copies of *CFHR3-CFHR1* (i.e., single dup) or 4 copies of *CFHR3-CFHR1* (i.e., double dup)*.* Vertical lines represent exons in a gene. Created in BioRender. Heiderscheit, A. (2025) https://BioRender.com/s87b337


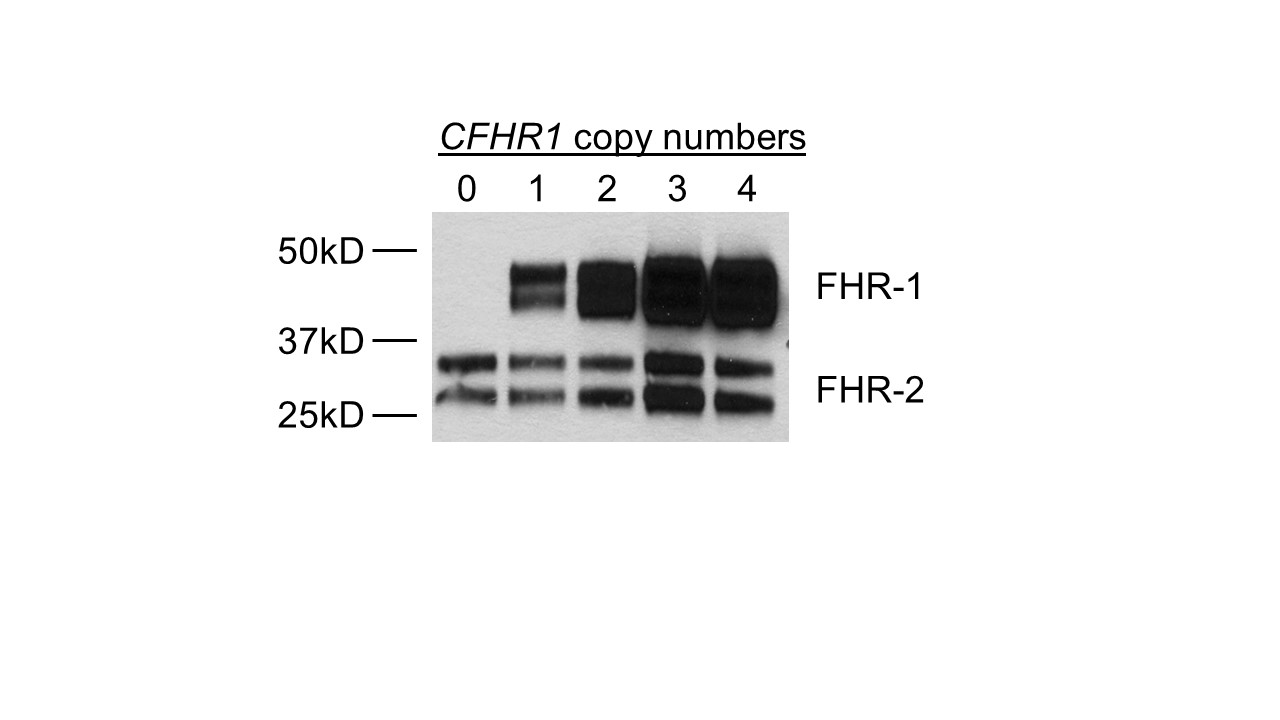


**Supplemental Figure 11. Copy numbers of *CFHR1****.* Factor H-related 1 (FHR-1) and Factor H-related 2 (FHR-2) protein levels in human serum with 0, 1, 2, 3, and 4 copies of *CFHR1.* FHR-1 and FHR-2 both circulate as two glycosylation variants represented by the double bands.


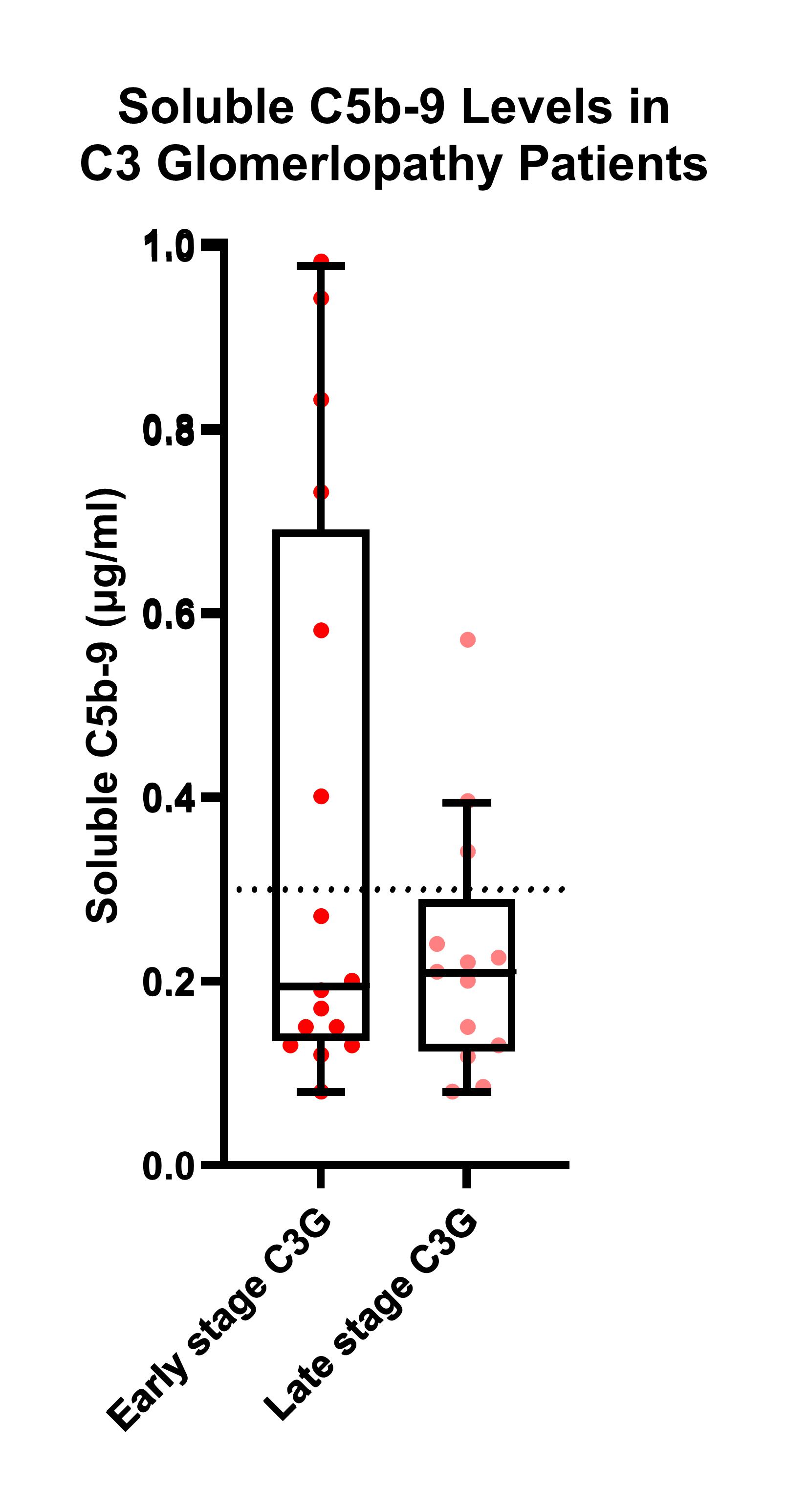


**Supplemental Figure 12. Soluble C5b-9 levels in C3 glomerulopathy patients with unknown drivers of disease***.* Soluble C5b-9 (sC5b-9) levels were not significantly different between early (n=16) and late (n=13) stage C3G patients with unknown drivers of disease. These data suggest that whilst an increased in the FHR-1/FH ratio as seen with declining renal function (see Figure 3) increases C3b deposition on cell deposition assays (see Figure 5), fluid-phase complement activity as measured by terminal complement complex formation is not increased (dashed line, normal range of sC5b-9 <0.3μg/ml; Mann-Whitney test).

**Supplemental Table 1. Characteristics of the chronic kidney disease controls**

| Characteristic | Number of people (%) or mean (SD) |
| --- | --- |
| Age | 64.2 (8.5) |
| Sex | 15 (30%) females  35 (70%) males |
| Diabetes | 20 (40%) |
| eGFR | 31.2 (7.4%) |
| CKD risk factor | |
| Hypertension | 17 (34%) |
| Diabetic nephropathy | 13 (26%) |
| Autosomal dominant polycystic kidney disease | 4 (8%) |
| Acute kidney injury | 5 (10%) |
| Interstitial disease/chronic obstruction | 7 (14%) |
| Solitary kidney | 2 (4%) |
| Focal segmental glomerulosclerosis | 1 (2%) |
| Unknown | 1 (2%) |
